# Berberine bridge enzyme‐like oxidases orchestrate homeostasis and signaling of oligogalacturonides in defense and upon mechanical damage

**DOI:** 10.1111/tpj.70150

**Published:** 2025-04-12

**Authors:** Ascenzo Salvati, Alessandra Diomaiuti, Federica Locci, Matteo Gravino, Giovanna Gramegna, Muhammad Ilyas, Manuel Benedetti, Sara Costantini, Monica De Caroli, Baptiste Castel, Jonathan D. G. Jones, Felice Cervone, Daniela Pontiggia, Giulia De Lorenzo

**Affiliations:** ^1^ Department of Biology and Biotechnologies 'Charles Darwin' Sapienza University of Rome Rome 00185 Italy; ^2^ Department of Biological and Environmental Sciences and Technologies University of Salento Campus Ecotekne Lecce 73100 Italy; ^3^ NBFC National Biodiversity Future Center Palermo 90133 Italy; ^4^ The Sainsbury Laboratory University of East Anglia, Norwich Research Park Colney Lane Norwich NR4 7UH UK; ^5^ Research Center for Applied Sciences for the Protection of the Environment and Cultural Heritage Sapienza University of Rome Rome Italy; ^6^ Present address: Department of Plant–Microbe Interactions Max‐Planck Institute for Plant Breeding Research Carl‐von‐Linné‐Weg 10 Cologne 50829 Germany; ^7^ Present address: Department of Crop Genetics John Innes Centre Norwich Research Park Norwich NR4 7UH UK; ^8^ Present address: Department of Environmental biology Sapienza University of Rome Rome 00185 Italy; ^9^ Present address: Department of Life, Health and Environmental Sciences University of L'Aquila L'Aquila 67100 Italy; ^10^ Present address: Institute of Nanotechnology, National Research Council (CNR‐NANOTEC) Campus Ecotekne Lecce 73100 Italy; ^11^ Present address: Laboratoire de Recherche en Sciences Vegetales (LRSV) Université de Toulouse, CNRS, UPS 24 chemin de Borde Rouge, Auzeville, BP42617 Castanet Tolosan 31326 France

**Keywords:** plant immunity, Damage‐Associated Molecular Patterns, oligogalacturonides, berberine bridge enzyme‐like oxidases

## Abstract

Plant immunity is triggered by endogenous elicitors known as damage‐associated molecular patterns (DAMPs). Oligogalacturonides (OGs) are DAMPs released from the cell wall (CW) demethylated homogalacturonan during microbial colonization, mechanical or pest‐provoked mechanical damage, and physiological CW remodeling. Berberine bridge enzyme‐like (BBE‐l) proteins named OG oxidases (OGOXs) oxidize and inactivate OGs to avoid deleterious growth‐affecting hyper‐immunity and possible cell death. Using OGOX1 over‐expressing lines and *ogox1/2* double mutants, we show that these enzymes determine the levels of active OGs vs. inactive oxidized products (ox‐OGs). The *ogox1/2*‐deficient plants have elevated levels of OGs, while plants overexpressing OGOX1 accumulate ox‐OGs. The balance between OGs and ox‐OGs affects disease resistance against *Pseudomonas syringae* pv. *tomato*, *Pectobacterium carotovorum*, and *Botrytis cinerea* depending on the microbial capacity to respond to OGs and metabolize ox‐OGs. Gene expression upon plant infiltration with OGs reveals that OGOXs orchestrate OG signaling in defense as well as upon mechanical damage, pointing to these enzymes as apoplastic players in immunity and tissue repair.

## Significance Statement

Homeostasis of damage‐associated molecular patterns (DAMPs) is essential to prevent harmful hyper‐immunity. OGOXs are enzymes that oxidize and inactivate in vitro the oligogalacturonides (OGs), i.e. the best characterized DAMPs in plants. By using both overexpressing and null mutants, we show that OGOXs regulate in planta the balance between active OGs and inactive oxidized OGs. The level of OGs activates critical defense genes and determines resistance against the bacteria *Pseudomonas syringae* pv. *tomato* and *Pectobacterium carotovorum*.

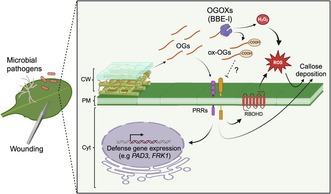

## INTRODUCTION

Plants have evolved, likely before animals, a sophisticated innate immune system to protect themselves against pathogens. During a pathogen attack, the recognition of danger signals occurs through specialized transmembrane receptors known as PRRs (pattern recognition receptors), which recognize a plethora of pathogenic signals known as microbe‐associated molecular patterns (MAMPs) and damage‐associated molecular Patterns (DAMPs) (Boller & Felix, 2009; De Lorenzo & Cervone, 2022; Molina et al., 2024).

Oligogalacturonides (OGs) are so far the best characterized DAMPs, deriving from the degradation of the plant cell wall (CW). They are oligomers of alpha‐1,4‐linked galacturonosyl residues released through the partial hydrolysis of homogalacturonan (HGA) that is a major component of pectin. OGs with a degree of polymerization (DP) between 10 and 15 show the highest immunity‐triggering activity (Degli Esposti et al., 2025; Ferrari et al., 2013; Pontiggia et al., 2024; Savatin et al., 2014) while shorter OGs may have a limited elicitor activity (Davidsson et al., 2017) or even work as repressors of immunity (Moerschbacher et al., 1999; Xiao et al., 2024).

The importance of OGs in plant signaling emerges not only in plant–microbe interactions but also in growth and development (Benedetti et al., 2015; Marfa et al., 1991; Mohnen et al., 1990; Spiro et al., 2002; Wolf, 2022). OGs antagonize auxin, positioning them as key regulators at the crossroads of development and defense (Branca et al., 1988; Savatin et al., 2011). Likely because signaling by OGs plays a key role *in planta*, their perception may involve multiple, partially redundant complexes, as proposed in Gravino et al. (2017), to make plants resilient to the loss of a primary signaling mechanism. WALL‐ASSOCIATED KINASE 1 (WAK1) has been proposed to participate in OG perception (Brutus et al., 2010), and possibly its paralogs (Kohorn et al., 2009). Contradictory results have been obtained in mutants lacking the entire *WAK* family (Anderson et al., 2001; Herold et al., 2024; Liu et al., 2023). Redundancy of the sensing systems and/or compensatory mechanisms, which have not been investigated yet, may explain why the mutants lacking the whole family still show responses to OGs (Blaschek, 2024). Indeed, recent findings highlight a more complex action of OGs in signaling than expected. They are major players in a newly discovered signaling mechanism that involves clustering of multiple types of receptors, including BRASSINOSTEROID INSENSITIVE 1 (BRI1) and FLAGELLIN‐SENSITIVE 2 (FLS2), and their global endocytosis. This process is triggered by the binding of OGs to RAPID ALKALINIZATION FACTOR (RALF), forming pectin‐RALF‐FERONIA‐LLG1 condensates (Liu et al., 2024).

Both pathogen‐ or plant‐secreted enzymes such as polygalacturonases (PGs) may catalyze the formation of OGs upon infection, wounding, or physiological cell wall remodeling (Ferrari et al., 2013; Savatin et al., 2014). An important notion introduced several years ago is that the release of elicitor‐active OGs from HG is not an event due to the casual loss of cell wall integrity but needs the concerted action and specific interaction of PGs with their plant‐derived protein inhibitors named PGIPs (Cervone et al., 1987, 1989). A recent work provides a structural demonstration that mechanistically validates this notion. The interaction between *Phaseolus vulgaris* PGIP2 (*Pv*PGIP2) and *Fusarium phyllophilum* PG (*Fp*PG) creates a substrate binding site on the *Pv*PGIP2‐*Fp*PG complex, assembling a new binary enzyme with a boosted substrate binding activity and altered substrate preference that preferentially produces long elicitor‐active OGs versus short immune‐repressive OGs (Xiao et al., 2024). The PG–PGIP interaction represents a unique plant mechanism to convert a pathogen virulence activity into a defense trigger and supports the view that OG signaling is an important component in plant immunity.

Analysis of the full‐genome expression reveals that OGs influence the expression of ~4000 genes in *Arabidopsis* (Ferrari et al., 2013). Accumulation of OGs increases plant resistance against pathogens, but their hyper‐accumulation results in plant growth penalties (Benedetti et al., 2015), reflecting the phenomenon known as growth–defense trade‐off (Huot et al., 2014; Pontiggia et al., 2020). The over‐accumulation of OGs can even lead to deleterious hyper‐immunity leading to cell death (Benedetti et al., 2015). A mechanism for controlling the homeostasis of OGs as well as other cell wall‐derived DAMPs such as cellulose and hemicellulose fragments may rely on specific oligosaccharide oxidases encoded by the berberine‐bridge enzyme–like (BBE‐l) gene family (Benedetti et al., 2018; Costantini et al., 2024; Locci et al., 2019). The BBE‐like protein family comprises 27 members (Daniel et al., 2015) and at least four members are capable of oxidizing OGs. These are OGOX1/At4g20830/BBE20, according to the nomenclature previously reported (Daniel et al., 2015), OGOX2/At4g20840/BBE21, OGOX3/At1g11770/BBE2, and OGOX4/At1g01980/BBE1 (Benedetti et al., 2018). Oxidation impairs the DAMP activity of OGs and produces H_2_O_2_ as a secondary product. In simultaneous co‐treatments, oxidized OGs did not interfere with the ability of OGs to up‐regulate the defense‐related genes *RetOx* (At1g26380/BBE3), also known as *FOX1* (Boudsocq et al., 2010) and *CYP81F2* (At5g57220) (Bednarek et al., 2009). Notably, in *Nicotiana benthamiana* agroinfiltrated leaves, the expression of OGOX1 inhibits the global receptor endocytosis mediated by OGs‐RALF‐FERONIA‐LLG1 condensates (Liu et al., 2024).

Expression of *OGOX1* is up‐regulated upon elicitation with OGs or bacterial MAMPs such as flg22 and elf18 (Boller & Felix, 2009), and upon pathogen infection in a strictly coordinated manner with *CELLOX1*, i.e., a BBE‐l protein capable of oxidizing cellulose fragments and mixed linked beta‐glucans (Costantini et al., 2024, Locci et al., 2019). Overexpression of *OGOX1* enhances the oxidation/inactivation of OGs but, nevertheless, enhances the resistance to the necrotroph *Botrytis cinerea*, due to the inability of the fungus to efficiently utilize the oxidized OGs as a carbon source (Benedetti et al., 2018). Whether OGOXs interfere also with OG‐mediated signaling in immune responses, however, has never been shown. We have used two bacterial pathogens in addition to *B. cinerea* and, through a reverse genetic approach, we demonstrate that OGOXs play a key role in the homeostasis of OGs in the apoplast and tune the immune responses.

## RESULTS

### OGOXs are apoplastic proteins

**Figure 1:**
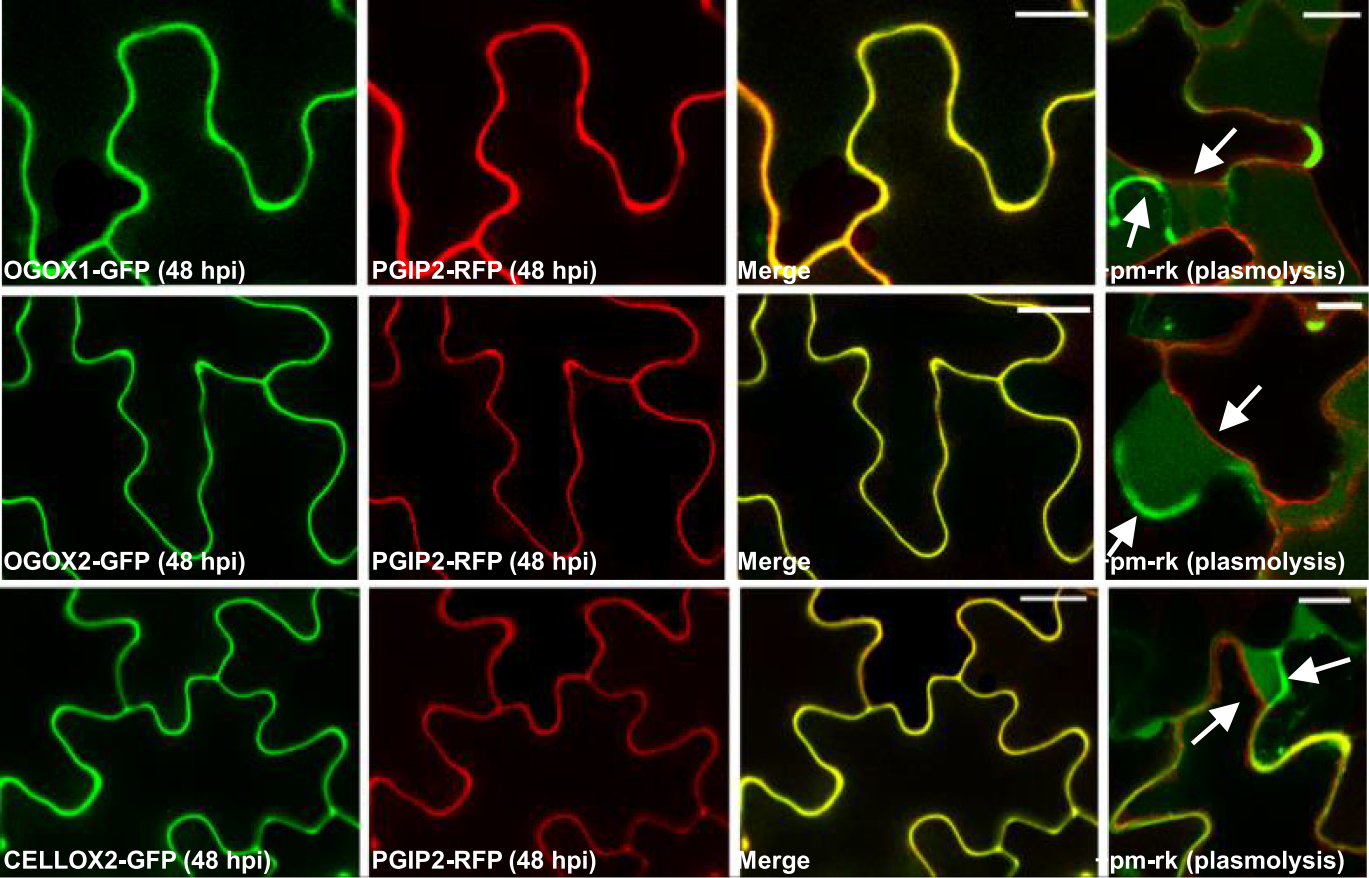
Transient expression of OGOX1‐GFP, OGOX2‐GFP, and CELLOX2‐GFP in tobacco epidermal cells. OGOX1‐GFP, OGOX2‐GFP, and CELLOX2‐GFP labeled the cell wall, colocalizing with the cell wall marker PGIP2‐RFP at 48 h post‐infiltration (hpi). Plasmolyzed cells coexpressing OGOX1‐GFP, OGOX2‐GFP, CELLOX2‐GFP, and the plasma membrane marker, pm‐rk, showed the presence of these proteins in the cell wall; the arrows evidence the green fluorescent cell wall and the retracted red fluorescent plasma membrane. Scale bars 20 μm for OGOX1‐GFP, OGOX2‐GFP, CELLOX2‐GFP, and PGIP2‐RFP; 10 μm for pm‐rk (plasmolysis).

Since the DAMP activity of OGs takes place in the apoplast, the proposed role of OGOXs in maintaining the OG homeostasis requires the demonstration that these enzymes are indeed localized in the apoplast. We therefore determined the subcellular localization of OGOX1 and its closest paralog OGOX2. Both enzymes possess a putative N‐terminal signal peptide for translocation into the endoplasmic reticulum and are predicted to be extracellular proteins, according to SIGNALP prediction. Both proteins, tagged with GFP at the C‐terminus, were transiently expressed in adult tobacco leaves by agroinfiltration and their co‐localization with the apoplastic RFP‐tagged *Phaseolus vulgaris* polygalacturonase‐inhibiting protein (*Pv*PGIP2) (De Caroli et al., 2015), was analyzed by confocal microscopy, also upon plasmolysis. As a control, CELLOX2, already shown to be apoplastic (Costantini et al., 2024), was analyzed in parallel. Both OGOX1 and OGOX2 clearly showed an apoplastic localization (Figure 1).

### *OGOX1* is transcriptionally up regulated during infection, wounding, and elicitor treatments

**Figure 2:**
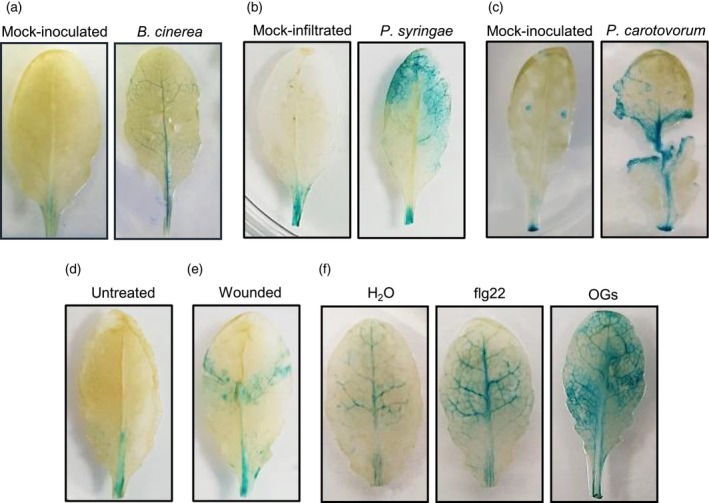
OGOX1::GUS transgenic leaves show increased GUS activity in response to pathogens, wounding and elicitor treatment. GUS activity was analyzed in OGOX1::GUS # 3.2 line. Results with a second independent line (#2.3) are in Figure S1. (a) Rosette leaves were drop‐inoculated with *B. cinerea* conidia (5 μl, ×10^5^ spore ml^−1^) or PDB (mock) as a control and GUS assay was performed 48 h post‐inoculation. (b) Rosette leaves were infiltrated with *P. syringae pv. tomato* DC3000 at OD = 0.002 or with H_2_O (mock‐infiltrated). GUS assay was performed 72 hpi. (c) Rosette leaves were drop‐inoculated at punctured sites with *P. carotovorum* cells (5 μl, OD_600_ = 0.025) or 50 mm potassium phosphate buffer pH 7.0 (mock‐inoculated). GUS assay was performed 14 hpi. (d) Untreated excised leaves analyzed at 72 h as a control. (e) Rosette leaves were wounded by crushing with knurled tweezers; GUS assay was performed 1 h after crushing. (f) Rosette leaves were infiltrated with OGs (200 μg ml^−1^), flg22 (100 nm), and water as control. GUS assay was performed 1‐h post‐infiltration.

The transcriptional regulation of *OGOX1* expression was analyzed in response to infection, wounding, and elicitor treatment in adult leaves, using two lines expressing the *GUS* reporter gene under the *OGOX1* promoter (OGOX1::GUS, lines #2.3 and #3.2; see molecular characterization in Figure S1a). *OGOX2* was not analyzed because it is not up‐regulated during the immune response (Benedetti et al., 2018). Upon infection with *B. cinerea*, histochemical staining showed that GUS activity is moderately induced in both OGOX1::GUS lines mainly in the vasculature (Figure 2a; Figure S1b). Upon infection with bacterial pathogens *Pseudomonas syringae* pv. *tomato* DC3000 (*Pst*) and *Pectobacterium carotovorum* (*Pc*), GUS activity was more vigorously induced in both lines (*P. syringae* pv. *tomato*, Figure 2b; Figure S1c and *P. carotovorum*, Figure 2c; Figure S1d). In the case of infection with *P. syringae* pv. *tomato*, there is a diffused and spread induction, whereas in the case of *P. carotovorum*, staining was observed around the damaged tissue and also at the cut site of the petiole in both infected and uninfected excised leaves, with some staining spreading to the proximal vasculature (Figure 2d; Figure S1e). Staining was also observed at the needle‐punctured uninfected sites (Figure 2c; Figure S1d), indicating an up‐regulation of *OGOX1* not only upon infection but also in response to mechanical damage. This was confirmed by the observed marked increase of GUS staining around the damaged tissue in leaves at 1 h after crushing (Figure 2e; Figure S1f). The response of both lines was also examined upon infiltration with either OGs or the MAMP flg22. Leaves showed intense GUS staining on the entire leaf area, including the vasculature, at 1 h post‐treatment, compared to control leaves infiltrated with water (Figure 2f; Figure S1g). Overall, these data indicate that *OGOX1* expression is induced upon infection, wounding, and treatment with elicitors.

### The expression of OGOX affects the levels of OGs and oxidized OGs (ox‐OGs) *in planta*

The impact *in planta* of an altered oxidation of OGs was studied in available over‐expressing lines of *OGOX1* (OGOX1‐OE #1.9 and #11.8) (Benedetti et al., 2018) and loss‐of‐function mutants, obtained in this work. In both overexpressing lines compared to the wild type (WT), transcripts of *OGOX1* were about 10 and 30 times higher, respectively (Benedetti et al., 2018). Besides *OGOX1*, OGOX2 also shows expression in leaves [Figure S2, data are from eFBrowser (Winter et al., 2007)], which is not inducible during the immune response (Benedetti et al., 2018). A contribution of *OGOX2* in concert with *OGOX1* to the homeostasis of OGs in leaves cannot, therefore, be excluded and we decided to generate an *ogox1/2* double loss‐of‐function mutant. As the two genes are closely linked on chromosome 4 (Benedetti et al., 2018) and cannot be separated by genetic crossing, double loss‐of‐function mutants were obtained by CRISPR/Cas9 genome editing (Figure S3a). Two *ogox1/2* double mutant lines (#1.5 and #4.6) were selected carrying frameshift mutations in both *OGOX1* and *OGOX2* in the homozygous state (Figure S3b).

**Figure 3:**
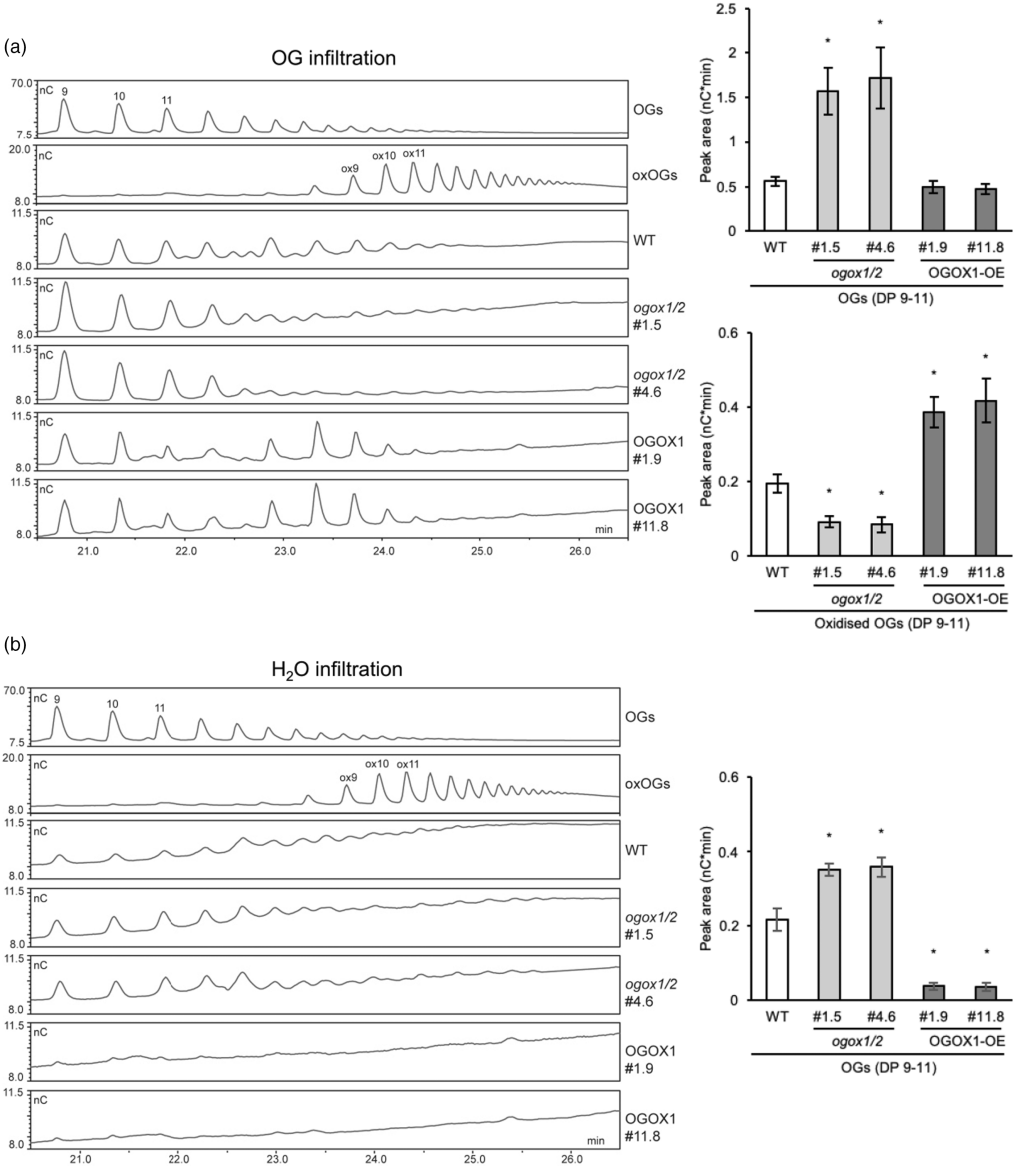
HPAEC‐PAD analyses of chelating agent‐extracted oligosaccharides (ChASS) from total cell wall preparations (AIS) of WT, *ogox1/2*, and OGOX1‐OE leaves. Leaves were infiltrated with (a) OGs (600 μg ml^−1^) or (b) water, and the fractions containing OGs (ChASS) were analyzed. In the chromatographic profiles (left), OGs and oxidized OGs (ox‐OGs) are indicated by numbers corresponding to the degree of polymerization (DP). Graphs on the right show the sum of peak areas of OGs and ox‐OGs (DP 9–11) as seen in the chromatographic profiles. No oxidized OGs were detected in water‐infiltrated leaves. OG and ox‐OG preparations of DP 9–16 were used as standards. Asterisks indicate statistical significant differences according to the student *t*‐test (*P < 0.05).

How oxidation affects the fate of both exogenous and endogenous OGs in adult leaves of the overexpressing lines and the double *ogox1/2* mutants was assessed. Levels of OGs and their oxidized counterpart (ox‐OGs) were determined by High‐performance anion‐exchange chromatography (HPAEC‐PAD) in the fraction indicated as chelating agent soluble solid (ChASS) from total CW preparations (alcohol insoluble solids [AIS]) of leaves from WT, OGOX1‐OE, and *ogox1/2* double mutants infiltrated with OGs or water (Pontiggia et al., 2015). Upon infiltration with OGs, higher levels of OGs and, conversely, lower levels of ox‐OGs were detected in the *ogox1/2* double mutants as compared with the WT. The opposite was found in the OGOX1‐OE plants (Figure 3a; Figure S4), indicating that upon infiltration, levels of OGOXs influence the relative balance between active OGs and ox‐OGs. The identity of the oligosaccharide fragments detected was confirmed by MALDI‐ToF analysis (Figure S6). Remarkably, in the water‐infiltrated leaves, we detected (i) higher levels of OGs in the *ogox1/2* mutants as compared with the WT, (ii) lower levels of OGs in the overexpressing plants, and (iii) no ox‐OGs in both OGOX1‐OE and *ogox1/2* plants (Figure 3b; Figure S5). In untreated, non‐infiltrated leaves, (basal) levels of OGs were similar in *ogox1/2* and WT plants but significantly lower in the case of OGOX1‐OE plants, whereas levels of ox‐OGs were undetectable in all genotypes (Figures S7 and S8). Overall, these results show that levels of OG oxidase activity control the level of OGs *in planta*.

### Expression of OGOXs affects defense‐related gene expression and callose deposition induced by OGs

**Figure 4:**
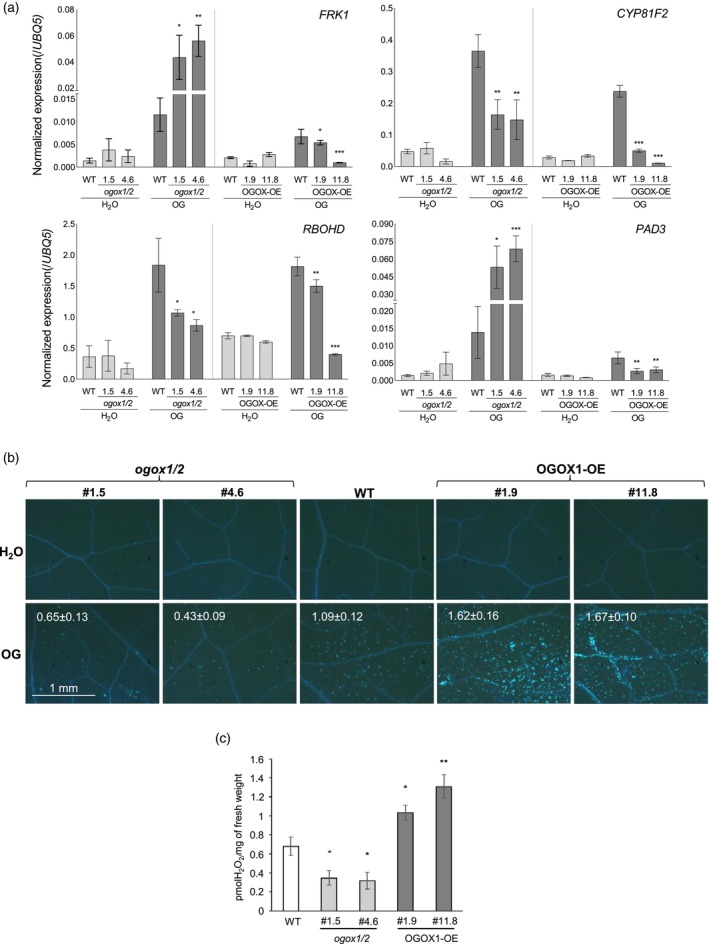
Altered expression of OGOXs affects defense‐related gene expression and callose deposition induced by OGs. (a) Expression was analyzed by quantitative RT‐PCR in rosette leaves from 4‐week‐old plants of *Arabidopsis* Col‐0 WT, OGOX1‐OE overexpressing plants (lines #1.9 and #11.8), and CRISPR Cas‐deleted *ogox1/ogox2* plants (lines #1.5 and #4.6) at 1 h (*FRK1*, *CYP81F2*, *RBOHD*) and 3 h (*PAD3*) post‐infiltration with OGs (60 μg ml^−1^) or water as control. *UBQ5* transcript levels were used for normalization. The mean of three biological replicates (±SD) is shown. To ensure accurate normalization, *UBC9* (At4g27960) was used as a second reference gene for *FRK1* and *CYP81F2* expression (Figure S9). (b) Callose deposition in WT, *ogox1/2*, and OGOX1‐OE leaves after infiltration with OGs and H_2_O. Callose deposits were stained with aniline blue 24 h after treatment. Each picture is representative of at least 18 pictures acquired per genotype per treatment. Callose deposits were counted using the “Analyze Particles” function of ImageJ. Values indicate fluorescent area/background ± SE. Values for callose deposition of *ogox1/2* and OGOX1‐OE lines were significantly different from WT, with a *P* < 0.05 as determined by Student's *t*‐test. (c) OG‐induced H_2_O_2_ accumulation in the culture medium of WT, *ogox1/2* and OGOX1‐OE seedlings. Measurements were taken 30 min after treatment with 30 μg ml^−1^ OGs. Values represent the mean of at least six replicates ± standard error (SE). Asterisks in (a, c) indicate statistically significant differences of mutants compared to WT treated plants according to Student's *t*‐test (*P < 0.05; **P < 0.01; ***P < 0.001).

Whether signaling by OGs is affected by their enzymatic oxidation was further investigated by analyzing gene expression in leaves of plants altered in OGOX levels, upon infiltration with OGs or water as a control. The following defense‐related genes as readouts of the OG signaling were examined: *FLG22‐INDUCED RECEPTOR‐LIKE KINASE 1* (*FRK1*), *CYP81F2*, *RESPIRATORY BURST OXIDASE HOMOLOG D* (*RBOHD*), analyzed at 1 h post‐infiltration, and *PHYTOALEXIN DEFICIENT 3* (*PAD3*) analyzed at 3 hpi (Figure 4; Figure S9) (Gravino et al., 2017; Locci et al., 2019; Manzoor et al., 2013). Upon infiltration with water, no significant difference in the expression of all marker genes was observed among the different genotypes (WT, *ogox1/2* and OGOX1‐OE) (Figure 4). However, levels of transcripts of all genes were higher in the water‐infiltrated leaves than in untreated non‐infiltrated leaves, indicating that infiltration upregulates their expression, in a similar way however in all of the genotypes (Figure S10). Upon infiltration with OGs, expression of the four genes was lower in the OGOX1‐OE plants than in the WT plants. (Figure 4a; Figure S9), a behavior that is expected if the levels of active OGs decrease due to the increased OGOX levels. Instead, in the *ogox1/2* mutants compared to the WT, expression of the four marker genes was differentially affected upon OG infiltration. Levels of *FRK1* and *PAD3* were higher, in agreement with the higher level of active OGs expected in the mutants. On the contrary, expression of *RBOHD* and *CYP81F2* was lower (Figure 4a; Figure S9); therefore, apparently not reflecting the increased levels of active OGs and suggesting a more complex impact of the lack of OGOX on immunity responses.

How alterations of OGOX expression affect a well‐known late response to elicitors, namely callose deposition (Wang et al., 2021), was also investigated. Fluorescence microscopy images (Figure 4b) of OG‐infiltrated leaves, compared to the WT, showed a lower deposition of callose after 18 h in the *ogox1*/2 mutants and a higher callose deposition in OGOX1‐OE lines. This finding, in line with the gene expression data, further suggests that not all the changes observed in OGOX‐manipulated plants can be explained solely by the levels of active OGs. Indeed, callose deposition has been shown to depend on the apoplastic H_2_O_2_ induced early after elicitation. For example, the lack of callose deposition observed in mutants lacking the apoplastic peroxidase PRX34 and the consequent apoplastic burst is rescued by adding H_2_O_2_ (Daudi et al., 2012). The action of OGOX, on the one hand, decreases the levels of elicitor‐active OGs but, on the other hand, increases the levels of H_2_O_2_ in the apoplast in the presence of the OGs as a substrate. It is likely, therefore, that the H_2_O_2_ generated by OGOX is responsible for the higher callose deposition in OGOX1‐OE lines. Indeed, OGOX1‐OE seedlings showed an increased H_2_O_2_ production compared to WT, whereas a significantly lower level of H_2_O_2_ was detected with the *ogox1/2* mutants. This may depend on the rapid action of OGOX present in the tissues on the added OGs (Figure 4c).

Collectively, our data indicate that the alterations of OGOX levels influence the signaling activity of OGs, likely involving H_2_O_2_ and possibly other unidentified factors that may modulate the plant defense.

### Basal resistance to pathogens is bent on the dynamics of OGs levels, their oxidation, and the microbial capacity of degrading ox‐OGs

**Figure 5:**
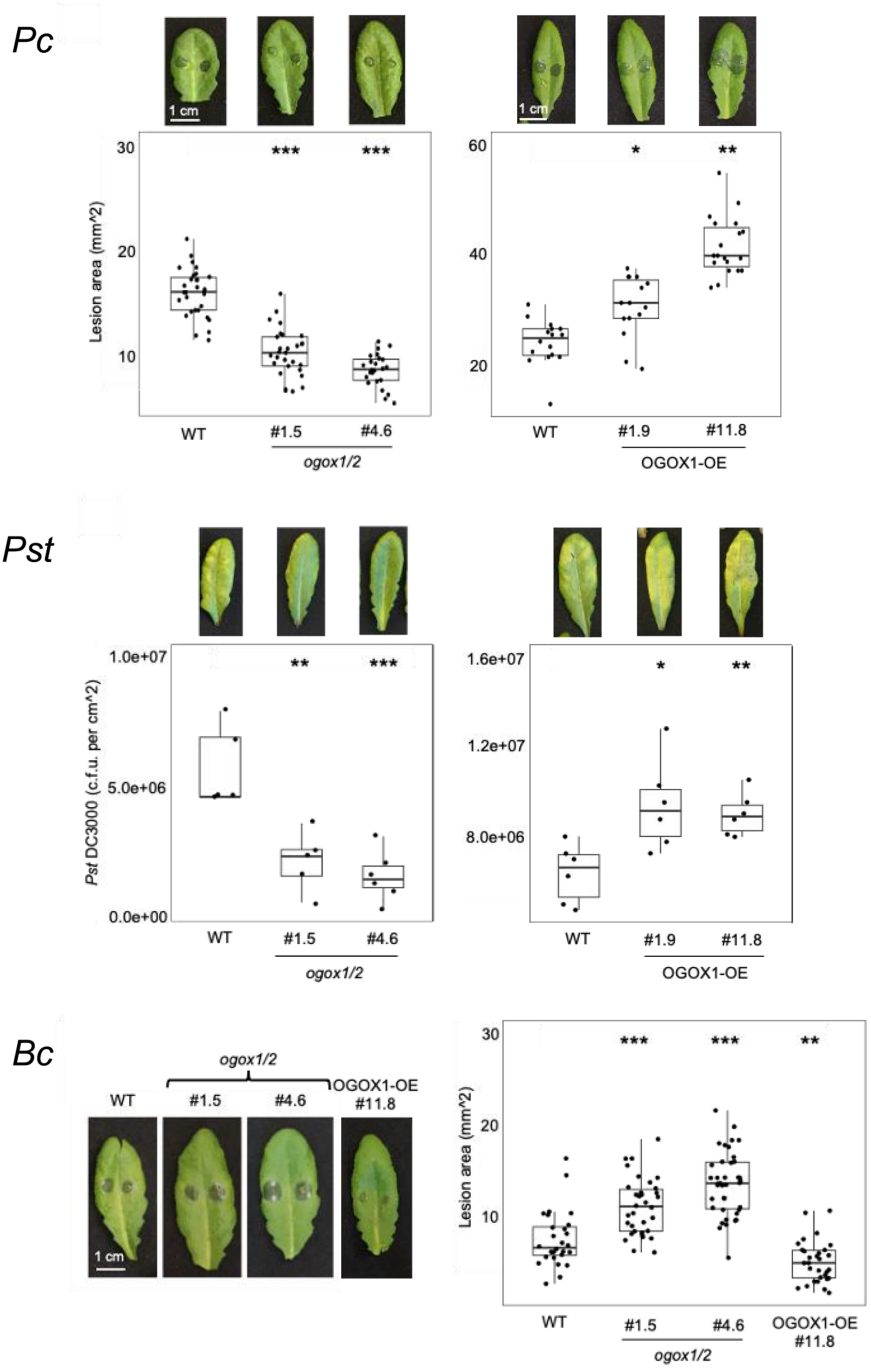
Plants altered in OGOX expression show altered response to pathogens. Lesion areas produced by *P. carotovorum* and *B. cinerea* were quantified at 16 and 48 hpi, respectively, using the ImageJ software. *Pseudomonas syringae* DC3000 (*P. syringae* pv. *tomato*) spread was quantified at 72 hpi. Asterisks indicate statistically significant differences of mutants compared to WT according to Student's *t*‐test (*P < 0.05; **P < 0.01; ***P < 0.001).

The above results clearly show that OGOXs have an impact on the signaling activity of OGs, which consequently may have an impact on the plant response to pathogens. To investigate this point, the response of plants altered in *OGOX* expression was monitored upon infection with the bacteria *P. syringae* pv. *tomato* and *P. carotovorum* and the fungus *B. cinerea*. Upon inoculation with either *P. syringae* pv. *tomato* or *P. carotovorum*, lesions measured at 16 and 72 h, respectively, were smaller in the *ogox1/2* mutants and larger in the OGOX1‐OE lines compared to the WT, indicating that oxidation of OGs reduces the plant resistance to these bacterial pathogens (Figure 5a,b). Opposite responses were observed upon inoculation with *B. cinerea*: the *ogox1/2* mutants showed a higher susceptibility and, conversely, the OGOX1‐OE plants exhibited increased resistance to *B. cinerea*. The last result confirms previously published data and can be explained at least in part by the reduced ability of this fungus to utilize ox‐OGs as a carbon source (Benedetti et al., 2018). On the other hand, the increased susceptibility of the *ogox1/2* mutants to *B. cinerea* suggests that the signaling activity of OGs in this interaction, unlike in the case of *P. syringae* pv. *tomato* and *P. carotovorum*, is marginal with respect to their role as a carbon source.

The utilization of ox‐OGs by *P. syringae* pv. *tomato* and *P. carotovorum* as a carbon source was next assessed by growing both bacteria, together with *B. cinerea* as a control, in a minimal medium containing OG or ox‐OGs, respectively. Unlike *B. cinerea*, which confirmed a reduced growth in the medium supplied with ox‐OGs, both *P. carotovorum* and *P. syringae* pv. *tomato* grew similarly in the media supplied either with ox‐OGs or OGs, indicating that both bacteria successfully metabolize both types of oligosaccharides as a carbon source (Figure S11). We concluded that the increased resistance of *ogox1/2* mutants and increased susceptibility of OGOX1‐OE plants to *P. carotovorum* and *P. syringae* pv. *tomato* depends on the relative amounts of elicitor‐active OGs *vs*. inactive ox‐OGs, unlike in the case of *B. cinerea*.

Because our results do not allow us to distinguish the individual roles of *OGOX1* and *OGOX2* in the response to pathogens, we also analyzed the contribution of *OGOX1* alone to the response to pathogens. A knock‐out T‐DNA insertional *ogox1* mutant was obtained that did not show detectable transcript levels and was therefore a null mutant (see characterization in Figure S12). Compared to the WT, the mutant showed increased susceptibility to *B. cinerea*, similar to what was observed in the *ogox1/2* mutants, whereas it showed no significant difference in its response to *P. syringae* pv. *tomato* and *P. carotovorum* (Figure S13a). These results indicate that only the simultaneous loss of *OGOX1* and *OGOX2* makes the plants more resistant to bacterial infection and suggests a redundant action of the two genes as susceptibility factors against bacterial pathogens, likely decreasing the levels of OGs in the infected tissues.

### Accumulation of ROS induced by mechanical damage increase in OGOX loss‐of‐function mutants

**Figure 6:**
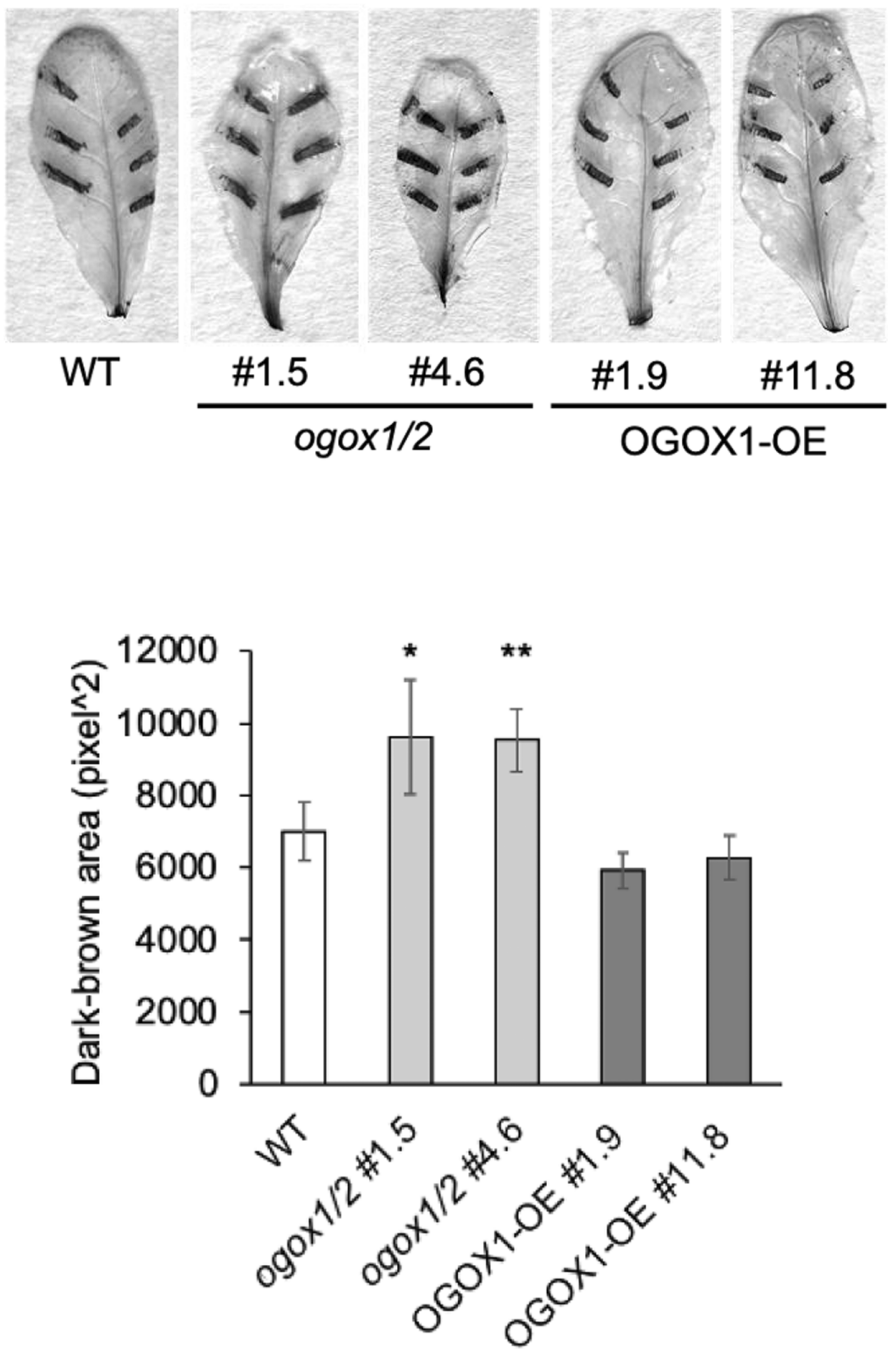
Hydrogen peroxide production in response to mechanical damage is higher in the *ogox1/2* mutants. Leaves of 4‐week‐old plants were damaged with knurled‐tip tweezers, excised after 1 h, and subjected to DAB staining for 4 h. *In situ* dark‐brown precipitate generated by DAB oxidation in the presence of hydrogen peroxide was quantified per leaf using the “Analyze Particles” function of ImageJ. Values are the mean ± SD of at least three damaged leaves from three different plants. The experiment was repeated three times with similar results; a representative experiment is shown. Asterisks indicate statistically significant differences of mutants compared to WT according to Student's *t*‐test (*P < 0.05; **P < 0.01).

OGs have been proposed to act as local signals in the response to wounding and tissue damage (De Lorenzo et al., 2018; Gramegna et al., 2016; Leon et al., 2001; Orozco‐Cardenas & Ryan, 1999; Ryan & Jagendorf, 1995; Savatin et al., 2014) and, indeed, in this work we show that *OGOX1* is transcriptionally up‐regulated around a wound site (Figure 2e; Figure S1f). We therefore investigated whether OGOXs play a role in the response to mechanical damage by measuring the hydrogen peroxide generated at mechanically injured sites. Crushing was inflicted by applying mild pressure to the leaf surface with forceps. Hydrogen peroxide production at damaged sites, detected by DAB staining, was increased in the *ogox1/2* mutants (Figure 6) as well as in the *ogox1* single mutants (Figure S13b) compared to WT plants. On the contrary, in the OGOX‐OE plants, no significant difference was observed.

## DISCUSSION

We show in this work that OGOXs regulate in planta the levels of OGs acting as DAMPs and influence their immunity‐related signaling and response to pathogens. Previous work demonstrated that overexpression of OGOX1 and, in a similar way, overexpression of CELLOX, which act on cellulose fragments and mixed linked beta‐glucans, makes plants more resistant to *B. cinerea*, at least in part because, remarkably, this fungus is unable to metabolize oxidized oligosaccharides as a carbon source (Benedetti et al., 2018; Costantini et al., 2024; Locci et al., 2019). Conversely, as shown here, *ogox1/2* double mutants are more susceptible to *B. cinerea*, reinforcing the previous result that overexpression of OGOX1 makes plants more resistant to *B. cinerea*. This result also suggests that resistance to *B. cinerea* is marginally correlated with the levels of active OGs and is primarily linked to their role as a carbon source. In the case of the bacterial pathogens *P. syringae* pv. *tomato* and *P. carotovorum*, which, unlike *B. cinerea*, grow equally well in the presence of OGs and ox‐OGs as a carbon source, instead we demonstrate that plant resistance does correlate with the levels of elicitor‐active OGs expected to be present in the plants altered in OGOX expression. Indeed, both bacteria have reduced ability to infect the *ogox1/2* double mutants, where the DAMP activity of OGs is higher, and increased ability to infect OGOX1‐overexpressing plants, where it is lower. Against these pathogens, both OGOX1 and OGOX2 appear to play a redundant role, as suggested by the wild‐type‐like behavior of the single mutant *ogox1*. Unlike *OGOX1*, *OGOX2* is not up‐regulated during the immune response and the encoded protein has been found to be subjected to post‐translational modifications (PTM) in etiolated seedlings, after the cleavage of the 26‐amino acid (aa) signal peptide for translocation into the ER. A new N‐terminus lacking the first amino acid of the mature protein (an alanine) was found, likely generated by endopeptidase cleavage (Zhang et al., 2018). Whether this or other kinds of PTM occur and modulate OGOX2 activity during immunity has never been investigated.

Taken together, our observations reinforce the notion that OGOXs, and likely other BBE‐l proteins, play an important role in governing the potential DAMP activity of plants during the immune response. They also strengthen the notion that OGs are important players in plant immunity.

The most obvious physiological role of OGOXs is the homeostatic control of OGs to prevent an excessive accumulation that provokes plant growth penalties. The homeostasis of OGs ensures the proper defense–growth trade‐off that maximizes the recovery of a plant attacked by a microbe (Benedetti et al., 2015). The requisites of OGOXs for such a role are evident in the finding that these enzymes are apoplastic proteins up‐regulated at the transcriptional level during pathogen attack, mechanical damage, and elicitor treatment, as shown in this paper. Alterations of their expression not only alter the fate of exogenous OGs in the expected direction but also influence the levels of OGs in untreated plants.

The orchestration of the OG levels by OGOX is evident in our gene expression analyses of the mutant/transgenic plants elicited with OGs. In the overexpressing plants, gene expression is affected in the expected direction towards an increased oxidation/inactivation of OGs. All the genes (*FRK1*, *CYP81F2*, and *RBOHD* examined at 1 h, and *PAD3* at 3 h) showed decreased expression due to the enzymatic oxidation of OGs impairing their signaling capability. In contrast, in the *ogox1/2* mutants, *FRK1* and *PAD3* exhibited the expected increased up‐regulation; however, *CYP81F2* and *RBOHD* showed reduced expression. This difference in gene expression behavior in the *ogox1/2* mutants suggests that, upon elicitation, the absence of OGOXs has a more complex impact on immunity‐related responses than can be explained solely by increased levels of active OGs. Noteworthy, both *RBOHD* and *CYP81F2* play an important role in elicitor‐induced callose deposition (Clay et al., 2009; Daudi et al., 2012), but our observations show no correlation between the transcript levels of these two genes and callose deposition. Transcripts were lower in *ogox1/2* plants in response to OGs and even lower in the OGOX1‐OE, while callose deposition was defective in the *ogox1/2* plants but increased in the OGOX1‐OE plants. Moreover, although callose deposition and expression of *RBOHD* and *CYP81F2* have been shown to be controlled by a peroxidase‐mediated apoplastic oxidative burst that enhances the activity of RBOHD establishing a feed‐forward loop for H_2_O_2_ accumulation (Daudi et al., 2012), in our assays we observed no correlation between regulation of *RBOHD* and *CYP81F2* and entity of the extracellular oxidative burst induced by the addition of OGs. Worth of note, this burst was lower in the *ogox1/2* plants and higher in the OGOX1‐OE plants, thereby showing no correlation with the levels of non‐oxidated active OGs expected to be present in these plants. This apparent inconsistency may depend on the fact that H_2_O_2_ is produced during oxidation of the OGs by the OGOXs and increases the levels measured in our experimental system. It remains to be elucidated whether and how OGOX‐produced H_2_O_2_ contributes to immunity signaling pathways and what impact OGOX activity, through its control of the active OGs/inactive ox‐OGs balance, has on other signaling pathways, such as those involving hormones.

Notably, upon a mechanical damage mainly consisting of a moderate crushing, and therefore in the absence of massive levels of exogenously added OGs, the *ogox1/2* and the *ogox1* mutants showed a higher accumulation of ROS at the injured site. This is likely due to the expected higher levels of OGs consequent to the lack of OGOXs around the wound site. In WT plants, tissue injury up‐regulates *OGOXs*, as shown by our observations in plants expressing the *OGOX1* promoter::GUS transcriptional fusion (see Figure 2), and may prevent an excessive DAMP activity of OGs, likely allowing the initiation of the repair process (Gramegna et al., 2016; Savatin et al., 2014). On the other hand, the up‐regulation of *OGOXs* at the injured site in WT plants may explain why there is no significant alteration in ROS accumulation in OGOX‐OE plants since a further increase in enzyme levels may have little effect on OGs and therefore ROS levels.

Taken together, our results show that OGOXs are important players in the response to both pathogens and mechanical damage. Furthermore, a recent study has illuminated a novel biological mechanism wherein the physical interplay between a fungal PG and a plant PGIP facilitates the generation of long OGs with DAMP activity (2024). This underscores that the generation of OGs is not a random occurrence but rather the outcome of a precise biological adaptation evolved by the plant to facilitate their release from the cell wall. In this paper, we reinforce the notion that specific mechanisms govern the levels of OGs *in planta* by demonstrating that OGs are subject to a homeostatic mechanism mediated by OGOX to regulate their activity.

Thus, the signaling activity of OGs, as previously discussed in many papers (De Lorenzo & Cervone, 2022; Degli Esposti et al., 2025; Pontiggia et al., 2020), increasingly emerges as “global” and relevant not only in immunity but also in growth and developmental processes. How the release of OGs is finely and differentially tuned by the plant cell in many physiological events and how their activity is orchestrated by OGOXs becomes a frontier topic in plant biology.

## MATERIALS AND METHODS

### Plant material and growth

*Arabidopsis thaliana* wild type (WT) ecotype Columbia (Col‐0) seeds were purchased from Lehle Seeds (Round Rock, TX, USA). The generation of OGOX1 overexpressing lines (OGOX1‐OE #1.9 and #11.8) has been described previously (Benedetti et al., 2018). The *ogox1* (WISCDSLOX432E05) T‐DNA insertional line (Woody et al., 2007) was obtained from the European Arabidopsis Stock Center (NASC). Plants were grown in a growth chamber at 22°C, 70% humidity, under irradiance of 100 μE m^−2^ sec^−1^ with a photoperiod of 12‐h light/12‐h dark.

### Generation of constructs for stable transformation of Arabidopsis and selection of transgenic plants

*Agrobacterium tumefaciens* strain GV3101 was transformed with recombinant plasmids by electroporation and used for stable transformation of Arabidopsis accession Col‐0, performed by the floral dip method (Clough & Bent, 1998).

To generate the OGOX1*::*GUS plants, the predicted promoter sequence of *OGOX1* was obtained from the Arabidopsis Gene Regulatory Information Server (AGRIS; arabidopsis.med.ohio‐state.edu/). A fragment corresponding to 3002 nucleotides upstream of the predicted translation codon start of *At4g20830* was amplified from Arabidopsis Col‐0 genomic DNA using the *BamHI*‐*POGOX1* Fwd and *BamHI*‐*POGOX1* Rev primers (Table S1). The promoter fragments were cloned in the binary vector pBI121 (Stratagene) by a two‐step reaction: (i) the CaMV 35S sequence was excised using the *Hin*dIII and *Xba*I restriction enzymes; (ii) the amplified fragment was cloned using the *Bam*HI restriction site of pBI121 upstream of the *uidA* gene. From 6 independent transformed plants, four T3 homozygous lines (OGOX1::GUS, lines #2, #3, #4, and #5) containing a single insertion carrying the OGOX1::GUS cassette were characterized for the levels of the transgene transcript (Figure S1). Two lines (#2 and #3) were selected for further analyses.

For genome editing of *OGOX1* and *OGOX2*, plasmids were assembled using the Golden Gate modular cloning method (Engler & Marillonnet, 2014). Five gRNA sequences for *OGOX1* and *OGOX2* (see Table S2) were chosen using “chopchop.rc.fas.harvard.edu” program. To generate the sgRNA expression cassettes, DNA fragments containing the ‘EF’ backbone with 67 bp U6‐26 terminator were amplified using primers flaked with BsaI restriction sites associated with Golden Gate compatible overhangs (see Table S1). The amplicons were assembled with the U6‐26 promoter (pICSL90002) in Level 1 vectors (see Table S2) according to the Golden Gate protocol. Briefly, 0.02 pmoles of purified PCR products were mixed with the same molar amount of the corresponding Level 1 and Level 0 (pICSL90002) vectors, 0.5 μl of BpiI enzyme (10 U μl^−1^; ThermoFisher Scientific, Waltham, MA, USA), 0.5 μl of T4 ligase (400 U μl^−1^; NEB), 1.5 μl of CutSmart Buffer (NEB), 10 × bovin serum albumin (1.5 μl) and water in a total reaction volume of 15 μl. The reaction was carried out as follows: 20 sec at 37°C, 25 cycles of (3 min at 37°C/4 min at 16°C), 5 min at 50°C and 5 min at 80°C. The resulting Level 1 vectors were then assembled in Level M vector (pAGM8055, Addgenie) following the same Golden Gate protocol. Cas9 expressing cassette with RPS5a promoter, the Cas9 coding sequence, and the E9 terminator, together with the FAST‐Red selectable marker (pBCJJ348) was generated by Castel et al. (2019). Combination of the two‐Level M vectors containing the sgRNAs and the Cas9 expression cassettes was assembled in Level P pICSL4723 (Addgenie) binary vector according to the Golden Gate protocol.

For selection of *OGOX1* and *OGOX2* FAST‐Red/Cas9/sgRNAs genome edited plants, 12 T1 transformed seeds (screened for the presence of the Fast‐Red selection marker by fluorescence stereoscopy) were germinated and grown (Figure S3). Mutations in both *OGOX1* and *OGOX2* were found in two plants, #1 and #4 (Figure S3). T2 Cas9‐free seeds without red fluorescence were selected, and single plants were genotyped to confirm the presence of the mutations seen in T1:T2 plants *ogox1/2* #1.5 and #4.6 showed frameshift mutations in homozygosis in both *OGOX1* and *OGOX2* (Figure S3). T3 and T4 seeds were obtained and genotyped, confirming the presence of the mutations in the homozygous state.

### Agroinfiltration and plasmolysis

Colocalization analyses were performed by *Agrobacterium tumefaciens‐*mediated transient expression in tobacco leaves. PGIP2‐RFP (De Caroli et al., 2011) and pm–rk (stock # CD3‐1007; http://www.bio.utk.edu/cellbiol/markers/) were used as a cell wall marker and as a plasma membrane marker, respectively. The 35S::OGOX1‐GFP [OGOX1.2 (Benedetti et al., 2018)] and 35S::OGOX2‐GFP constructs, with both OGOX1 and OGOX2 tagged with GFP at the C‐terminus, were generated using the Gateway System vector (Thermo Fisher Scientific). The 35S::CELLOX1‐GFP construct was obtained using the Gateway system vectors, pDONR221 (Invitrogen) and pK7FWG2 (V141), using eGFP as a C‐terminal tag (Karimi et al., 2002). The 35S::CELLOX2‐GFP construct has been previously described (Costantini et al., 2024).

The infiltration buffer (50 mm MES, pH 5.6, 2 mm sodium phosphate buffer, pH 7.0, 100 μm acetosyringone, 0.5% glucose) was used to resuspend the transformed *A. tumefaciens* for inoculation. OD at 600 nm was measured to have a final value of 0.5 for each sample. For co‐transformation, *Agrobacterium* cultures were grown separately and mixed prior to the infiltration of *N. tabacum* leaves using a needless syringe. The infiltrated leaves were left for 48 h in the growth chamber before confocal microscope visualization, using a Zeiss LSM 710 microscope. For plasmolysis experiments, leaf disks at 2 days post‐infiltration were treated with 1 m NaCl, incubated for 10 min, and observed at the confocal microscope as previously described (De Caroli et al., 2014).

### Plant treatments

For GUS activity analysis in OGOX1::GUS plants, treatments were performed as follows. For the response to elicitors, adult leaves were infiltrated with water, flg22 (100 nm) or OGs solution (200 μg ml^−1^) using a needleless syringe and, after 1 h, were detached from plants and the assay was performed. For the response to wounding, adult leaves were wounded by applying a single pressure on each side of the lamina flanking the middle vein with laboratory forceps. After 1 h, leaves were detached from plants and the GUS assay was performed. Infections were performed as described below.

Gene expression was analyzed by quantitative RT‐PCR (Supporting Information: Materials and Methods) in rosette leaves of 4‐week‐old plants at 1 h (*FRK1*, *CYP81F2*, *RBOHD*) and 3 h (*PAD3*) post‐infiltration with OGs (60 μg ml^−1^) or water as control.

Callose deposition was detected in leaves of 4‐week‐old plants infiltrated with a solution containing OGs at a final concentration of 60 μg ml^−1^; detection was performed 24 h post‐infiltration.

OG‐induced H_2_O_2_ accumulation in the culture medium of seedlings was analyzed by the Xylenol Orange Assay 30 min after treatment with 30 μg ml^−1^ OGs.

### GUS analyses

Histochemical staining for GUS activity was performed by incubating leaves in the staining buffer (0.5 mg ml^−1^ X‐Glca [5‐bromo‐4‐chloro‐3‐indolyl β‐d‐glucuronic acid cyclohexylammonium salt; Duchefa Biochemie]; 2 mm K_3_[Fe(CN)_6_]; 2 mm K_4_[Fe(CN)_6_]; 0.2% Triton X‐100; 50 mm buffer sodium phosphate, pH 7.2; 2% DMSO) overnight at 37°C, with shaking. Subsequently, the samples were cleared and dehydrated with 100% boiling ethanol and rehydrated in 50% ethanol before photography.

### Infection assays

*Botrytis cinerea* conidia were suspended in potato dextrose broth (PDB, 24 g L^−1^; Difco, Detroit, MI, USA) at a final concentration of 5 × 10^5^ conidia ml^−1^ and incubated for 2–3 h at room temperature before inoculation of Arabidopsis leaves as previously described (Giovannoni et al., 2021). Adult leaves were inoculated with 5‐μl drops of a conidiospore suspension (5 × 10^5^/mL) in PDB or PDB only (mock), regularly spaced on each side of the middle vein and, after 48 h, leaves were detached for analysis (GUS staining or symptom analysis).

*Pectobacterium carotovorum* subsp. *carotovorum* strain DSMZ 30169 (*P. carotovorum*) was grown as previously described (Gramegna et al., 2016). For inoculation, adult leaves were punctured with the sterile needle of a 1‐ml syringe on the epidermis of the adaxial surface of each leaf, at the sides of the middle vein, and a 5‐μl droplet of the bacterial suspension (OD_600_ = 0.05) or mock (50 mm potassium‐phosphate buffer, pH 7.0) was placed on each punctured site. Leaves were excised after 16 h for GUS staining. Symptoms caused by *B. cinerea* and *P. carotovorum* were assessed by measuring the area of macerated tissue (lesion area), at 48–and 16‐h post‐inoculation respectively, using ImageJ software. (https://imagej.nih.gov/ij/).

*Pseudomonas syringae* pv. *tomato* (*P. syringae* pv. *tomato*) DC3000 was cultured in LB liquid medium containing 25 μg ml^−1^ rifampicin, and inoculations were performed by infiltrating adult leaves with water or the bacterial suspension (5 × 10^4^ cfu ml^−1^) using a needleless syringe as previously described (Böhm et al., 2014). GUS staining was performed after 3 days. Bacterial growth was quantified at 0‐ and 3‐days post infiltration.

### Preparation of OGs and oxidized OGs

The oligogalacturonide (OG) standard preparation was the same as in a previous work (Bigini et al., 2024) and was prepared by partial digestion of purified polygalacturonic acid (PGA) using endo‐polygalacturonase II from *Aspergillus niger*. The ox‐OGs were obtained by incubating the standard OGs with OGOX1 expressed in *Pichia pastoris*, as previously described (Benedetti et al., 2018), and their oxidation was assessed by both HPAEC‐PAD (Fig. 3, Fig. S4) that shows the retention time shift in the chromatographic profiles, as previously reported by Benedetti et al. (2018) and mass spectrometry (Fig. S6B). The retention time shift was approximately 3 min for the nonamer.

### Analysis of OGs and oxidized OGs in Arabidopsis plants by HPAEC‐PAD (high‐performance anion‐exchange chromatography with a pulsed amperometric detector)

Two leaves (about 100 mg of fresh weight) of 4‐week‐old plants were each infiltrated, using a syringe without the needle, with 200 μL sterile ultrapure water or with 600 ng μl^−1^ OGs. Fifteen min after infiltration, leaves were excised, frozen in liquid nitrogen, and homogenized for 2 min at 30 Hz in a mixer mill MM301 (RETSCH), using inox beads (6 mm diameter). To isolate cell wall polysaccharides that are AIS, ground tissues were re‐suspended in 1 ml of 70% v/v ethanol pre‐warmed to 70°C. The pellet was washed twice with a chloroform:methanol (1:1, vol/vol) mixture, vortexed, and centrifuged at 14 000 ***g*** for 10 min. Following this, it was washed twice with acetone and centrifuged at 14 000 ***g*** for 10 min. Finally, the pellet was dried at room temperature under a chemical hood overnight. For extraction of the Chelating Agent Soluble Solids (ChASS) fraction containing OGs, AIS fractions were re‐suspended in 200 μl chelating solution 2 (ChA2: 50 mm CDTA, 50 mm ammonium oxalate, 50 mm ammonium acetate, pH 5.5) and incubated overnight at 4°C as detailed (Pontiggia et al., 2015). The ChASS fraction was then precipitated with 20% ethanol and, after re‐suspension in ultrapure water, it was analyzed using HPAEC‐PAD with a CarboPac PA200 3 × 250 mm analytical column with a guard column (Thermo Fisher), as previously described (Benedetti et al., 2017) (Figure 3; Figures S4 and S5). ChASS fractions from untreated leaves have been analyzed by CarboPac PA100 3 × 250 mm analytical column with a guard column (Thermo Fisher) kept at 35°C. The flow rate was 0.4 ml min^−1^ and eluents A (0.05 m NaOH) and B (1 m Na‐acetate in 0.05 m NaOH) were applied after injection as follows: 20% B for 45 min followed by 70% B for 5 min and 90% B for 5 min, 20% B for 10 min for the equilibration (Figures S7 and S8).

### Mass spectrometry analysis

Mass spectrometry analysis was conducted as described in a previous work (Bigini et al., 2024). In particular, MALDI‐ToF MS measurements were performed using an UltrafleXtreme TOF/TOF mass spectrometer equipped with a reflector and controlled by the FlexControl 2.2 software package (Bruker Daltonics).

### Gene expression analysis: RNA extraction, RT reaction, and real‐time RT‐PCR

Total RNA was extracted using RNA isolation NucleoZol (Macherey‐Nagel) according to the manufacturer's instructions and treated with RQ1 RNase‐free DNase (Promega). cDNA was synthesized with ImProm‐II™ Reverse Transcription System (Promega). qRT‐PCR was performed with a CFX96 Real‐Time PCR System (BioRad) using iTaq Universal SYBR Green Supermix (BioRad) as recommended by the manufacturer. The amplification protocol consisted of 30 sec of initial denaturation at 95°C, followed by 45 cycles of 95°C for 15 sec, 58°C for 15 sec, and 72°C for 15 sec. Primers used for gene expression analysis are listed in Table S1. At least three standard curves were run for each primer pair, and the average qPCR efficiency was calculated using the equation: *E* = (10^−1/slope^ – 1) × 100 (where *E* is qPCR efficiency, and the slope is the gradient of the standard curve). The expression levels of each gene were determined relative to *AtUBQ5*, using a modification of the Pfaffl method (Pfaffl, 2001). A second reference gene, *UBC9* (AT4G27960), was also used for some genes (*FRK1* and *CYP81F2*) to ensure accuracy. Average cycle threshold (CT_a_) was calculated for each gene from three replicates, corrected for the efficiency (*E*) of PCR as follows: CT_a_ × log_2_(1 + *E*). ΔCT between genes and reference genes was then calculated for each sample, and expression levels for each gene transcript were calculated as 2^−ΔCT^ and were expressed in arbitrary units.

### Pathogen growth on OGs and oxidized OGs

Growth assays of *B. cinerea* on OGs and oxidized OGs were done as previously described (Benedetti et al., 2018). *P. carotovorum* subsp. *carotovorum* and *P. syringae* DC3000 were grown in a minimal medium [13 mm potassium phosphate pH 7, 17 mm NaCl, 1.7 mm sodium citrate, 30 mm (NH_4_)_2_SO_4_ and 2.8 mm MgSO_4_] supplemented with the appropriate carbon source. Bacterial growth was carried out on a rotary shaker (160 rpm) at 28°C using 1.4 × 10^7^ cell ml^−1^ as the starting inoculum. Growth was determined spectrophotometrically by measuring the optical density at 600 nm (OD_600_) after 24 h of incubation. Three replicates for each growth were performed. For the growth of all three pathogens in the presence of OGs or ox‐OGs, the minimal medium was supplied with OGs or ox‐OGs with a degree of polymerization ranging from 4 to 10 at a final concentration of 0.15% (w/v) before filter‐sterilization. Pathogen growth (%) was calculated as the percentage ratio between the growth measured in the presence of OGs or ox‐OGs and that in minimal medium supplemented with 0.15% (w/v) d‐glucose.

### Callose deposition assays

Callose deposition was detected as previously described with some modifications (Galletti et al., 2008). Leaves of 4‐week‐old plants were infiltrated with a solution containing OGs at a final concentration of 60 μg ml^−1^; detection was performed 24 h post‐infiltration. After staining, leaves were mounted in 50% (v/v) glycerol and examined using a UV epifluorescence microscope (Nikon; Eclipse E200) equipped with a cooled charge‐coupled device camera (DS‐Fi1C). Images were acquired with the Nis Elements AR software (Nikon). Callose deposits were counted using the “analyze particles” function of ImageJ.

### Xylenol orange assay

Extracellular H_2_O_2_ by OG‐treated 13‐days‐old seedlings was measured by a colorimetric method (Gay et al., 1999) based on the peroxide‐mediated oxidation of Fe^2+^, followed by the reaction of Fe3^+^ with the xylenol orange dye (*o*‐cresolsulfonephthalein 3′,3′′‐bis[methylimino] diacetic acid, sodium salt; Sigma). Ten seeds were placed in each well of 12‐well plates containing 2 ml of liquid MS/2 medium. At least nine biological replicates for each genotype for the treatment were set up. One day before treatment, the medium was refreshed and leveled off. The treatment was performed by adding an OG solution to the medium to reach a final OG concentration of 30 μg ml^−1^. H_2_O was used as a control. Thirty minutes post treatment, 100 μl of medium was collected and 100 μl of xylenol orange final reagent solution was added. Standard curves of H_2_O_2_ were obtained for each independent experiment. Absorbance (*A*_560_) of the Fe^3+^‐xylenol orange complex was detected by a microplate reader (BioRad). Data were normalized and expressed as picomolar H_2_O_2_/mg fresh weight of seedlings.

### DAB staining upon mechanical damage

H_2_O_2_ visualization by DAB staining was performed basically as already described (Daudi et al., 2012). Briefly, mechanical damage was inflicted to leaves of 4‐week‐old Arabidopsis plants by applying a single mild pressure on each side of the lamina flanking the middle vein with a knurled‐tip tweezer (six lesions per leaves). After 1 h, leaves were detached from plants and dipped for 4 h in a solution containing 1 mg ml^−1^ 3,3‐diaminobenzidine (DAB) pH 5.0 at room temperature, with shaking. Subsequently, the samples were cleared and dehydrated with 100% boiling ethanol and rehydrated in 50% ethanol before taking pictures. *In situ* dark‐brown precipitate generated by DAB oxidation in the presence of H_2_O_2_ was quantified per leaf using the “Analyze Particles” function of ImageJ. Dark‐brown spots not generated by the tweezers, such as those at the excision site or in the central vein, or spurious spots, were excluded from the analysis.

## ACCESSION NUMBERS

At4g20830, At4g20840.

## AUTHOR CONTRIBUTIONS

AS and AD carried out most of the experiments and data analysis. FL, MG, GG, DP, MB, SC, MDC, MI, and BC performed the experiments. DP, GDL, and JDGJ supervised the experiments. FC and GDL wrote the manuscript draft, whereas FC, DP, and GDL edited the final version of the manuscript. All authors have approved the final manuscript.

## CONFLICT OF INTEREST

The authors declare no conflict of interest.

## Supporting information


**Figure S1.** Characterization of the *POGOX1::GUS* transgenic plants.
**Figure S2.** OGOX2 (At4g20840) is expressed in leaves.
**Figure S3.** Construction strategy for *OGOX1* and *OGOX2* gene editing by CRISPR/Cas9 via *Agrobacterium*‐mediated transformation of Arabidopsis and mutant screening approaches.
**Figure S4.** Complete HPAEC‐PAD chromatographic profiles of chelating agent‐extracted oligosaccharides (ChASS) from total cell wall preparations (AIS) of WT, *ogox1/2*, and OGOX1‐OE leaves infiltrated with OGs.
**Figure S5.** Complete HPAEC‐PAD chromatographic profiles of chelating agent‐extracted oligosaccharides (ChASS) from total cell wall preparations (AIS) of WT, ogox1/2, and OGOX1‐OE leaves infiltrated with H_2_O.
**Figure S6.** MALDI‐TOF full‐scan mass spectrum (MS) of chelating agent‐extracted oligosaccharides (ChASS) from total cell wall preparations (AIS) of WT, *ogox1/2*, and OGOX1‐OE adult leaves infiltrated with OGs.
**Figure S7.** HPAEC‐PAD analyses of chelating agent‐extracted oligosaccharides (ChASS) from total cell wall preparations (AIS) of WT, *ogox1/2*, *and OGOX1‐OE leaves*.
**Figure S8.** Complete HPAEC‐PAD chromatographic profiles of chelating agent‐extracted oligosaccharides (ChASS) from total cell wall preparations (AIS) of WT, *ogox1/2*, and OGOX1‐OE leaves.
**Figure S9.** Quantitative RT‐PCR analysis of *FRK1* and *CYP81F2* expression induced by OGs using two different housekeeping genes as reference (*UBQ5* and *UBC9*).
**Figure S10.** Analysis of defense‐related gene expression in untreated non‐infiltrated plants and plants infiltrated with water.
**Figure S11.** OG‐to‐growth conversion by *B. cinerea*, *P. carotovorum*, and *P. syringae*.
**Figure S12.** Characterization of the T‐DNA insertional mutants *ogox1*.
**Figure S13.** Analysis of pathogen resistance and hydrogen peroxide accumulation induced by mechanical damage in the *ogox1* null mutant and in the OGOX1‐overexpressing lines.
**Table S1.** Primers used in this work.
**Table S2.** sgRNAs targeting sequences and Level 1 plasmids.

## Data Availability

All data needed to evaluate the conclusions in the paper are present in the paper and/or the Supporting Information. Request for plant materials should be submitted to G.D.L.
