# Supplementary material for: Berberine bridge enzyme‐like oxidases orchestrate homeostasis and signaling of oligogalacturonides in defense and upon mechanical damage
Source: Plant J. 2025 Apr 12;122(1):e70150. doi: 10.1111/tpj.70150 (PMC11992967; doi:10.1111/tpj.70150)
Supplement: Supplementary file 1 — Figure S1. Characterization of the POGOX1::GUS transgenic plants. Figure S2. OGOX2 (At4g20840) is expressed in leaves. Figure S3. Construction strategy for OGOX1 and OGOX2 gene editing by CRISPR/Cas9 via Agrobacterium‐mediated transformation of Arabidopsis and mutant screening approaches. Figure S4. Complete HPAEC‐PAD chromatographic profiles of chelating agent‐extracted oligosaccharides (ChASS) from total cell wall preparations (AIS) of WT, ogox1/2, and OGOX1‐OE leaves infiltrated with OGs. Figure S5. Complete HPAEC‐PAD chromatographic profiles of chelating agent‐extracted oligosaccharides (ChASS) from total cell wall preparations (AIS) of WT, ogox1/2, and OGOX1‐OE leaves infiltrated with H2O. Figure S6. MALDI‐TOF full‐scan mass spectrum (MS) of chelating agent‐extracted oligosaccharides (ChASS) from total cell wall preparations (AIS) of WT, ogox1/2, and OGOX1‐OE adult leaves infiltrated with OGs. Figure S7. HPAEC‐PAD analyses of chelating agent‐extracted oligosaccharides (ChASS) from total cell wall preparations (AIS) of WT, ogox1/2, and OGOX1‐OE leaves. Figure S8. Complete HPAEC‐PAD chromatographic profiles of chelating agent‐extracted oligosaccharides (ChASS) from total cell wall preparations (AIS) of WT, ogox1/2, and OGOX1‐OE leaves. Figure S9. Quantitative RT‐PCR analysis of FRK1 and CYP81F2 expression induced by OGs using two different housekeeping genes as reference (UBQ5 and UBC9). Figure S10. Analysis of defense‐related gene expression in untreated non‐infiltrated plants and plants infiltrated with water. Figure S11. OG‐to‐growth conversion by B. cinerea, P. carotovorum, and P. syringae. Figure S12. Characterization of the T‐DNA insertional mutants ogox1. Figure S13. Analysis of pathogen resistance and hydrogen peroxide accumulation induced by mechanical damage in the ogox1 null mutant and in the OGOX1‐overexpressing lines. Table S1. Primers used in this work. Table S2. sgRNAs targeting sequences and Level 1 plasmids. [file TPJ-122-0-s001.pdf]

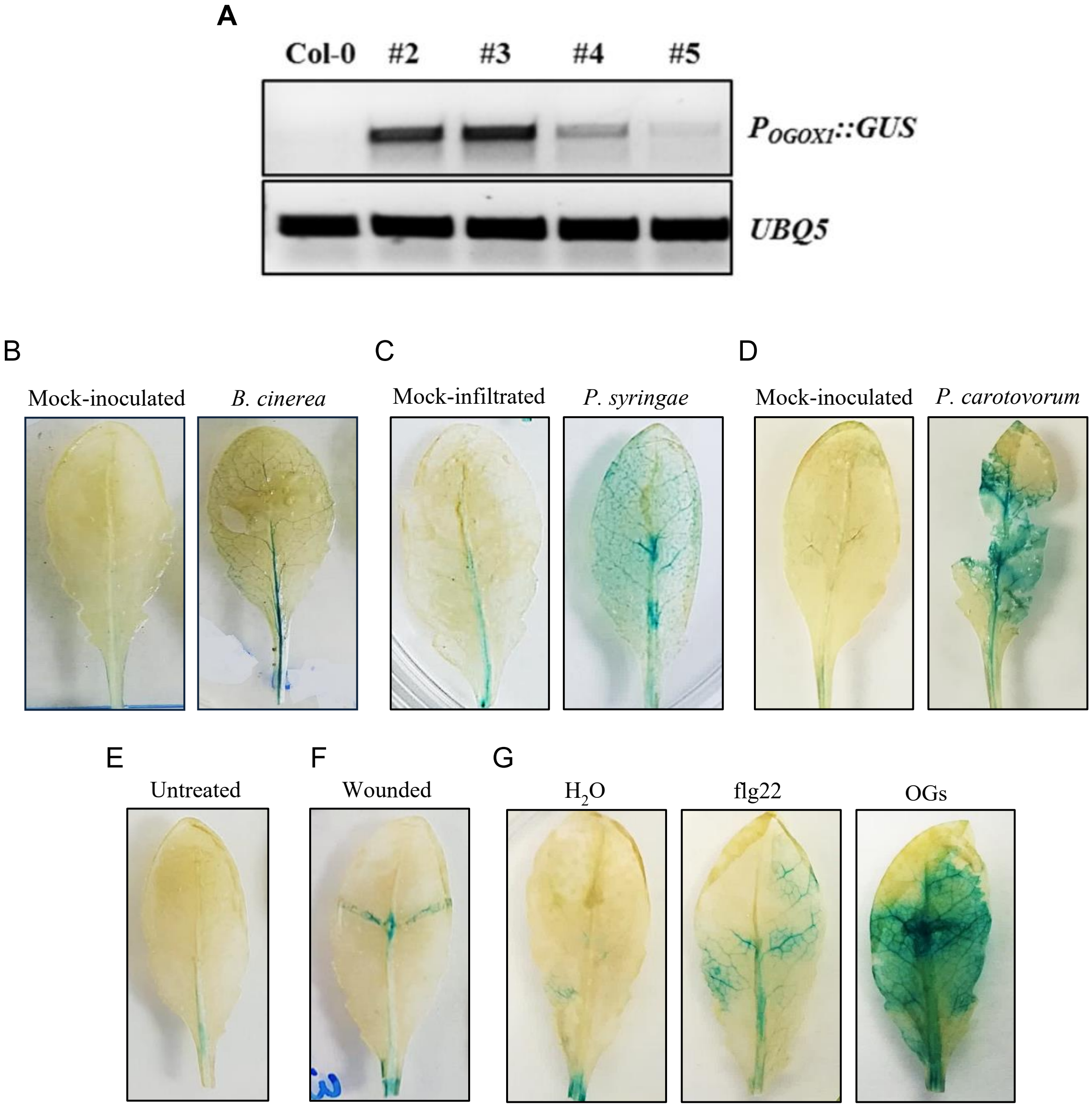

Figure S1. Characterization of the *POGOX1::GUS* transgenic plants.
A. The expression of the *POGOX1::GUS* transgene was analyzed by semiquantitative RT-PCR in wild-type (Col-0) and in four lines showing a 3:1 segregation of the insertion, which indicates a single insertion. The analysis was performed in T2 transgenic seedlings, using *POGOX1* and *GUS* specific primers. The lines #2 and #3 were chosen for further analyses. **B-D**.The OGOX1::GUS transgenic line # 2.3 line leaves show increased GUS activity in response to pathogens, wounding and elicitor treatment. **B.** Rosette leaves were drop-inoculated with *B. cinerea* conidia (5 µl, x10^5spore/ml) or PDB (mock) as a control and GUS assay was performed 48 hours post-inoculation. **C)** Rosette leaves were infiltrated with *P. syringae pv. tomato (Pst)* DC3000 at OD=0.002 or with H_2O (mock-infiltrated). GUS assay was performed 72 hpi. **D.** Rosette leaves were drop-inoculated at punctured sites with *P. carotovorum* cells (5 µl, OD600=0.025) or 50 mM potassium phosphate buffer pH 7.0 (mock-inoculated). GUS assay was performed 14 hpi. **E.** Untreated excised leaves analyzed at 72 h as a control. **F.** Rosette leaves were wounded by crushing with knurled tweezers; GUS assay was performed 1 hour after crushing. **G.** Rosette leaves were infiltrated with OGs (200 µg/ml), flg22 (100 nM) and water as control. GUS assay was performed 1-hour post-infiltration.

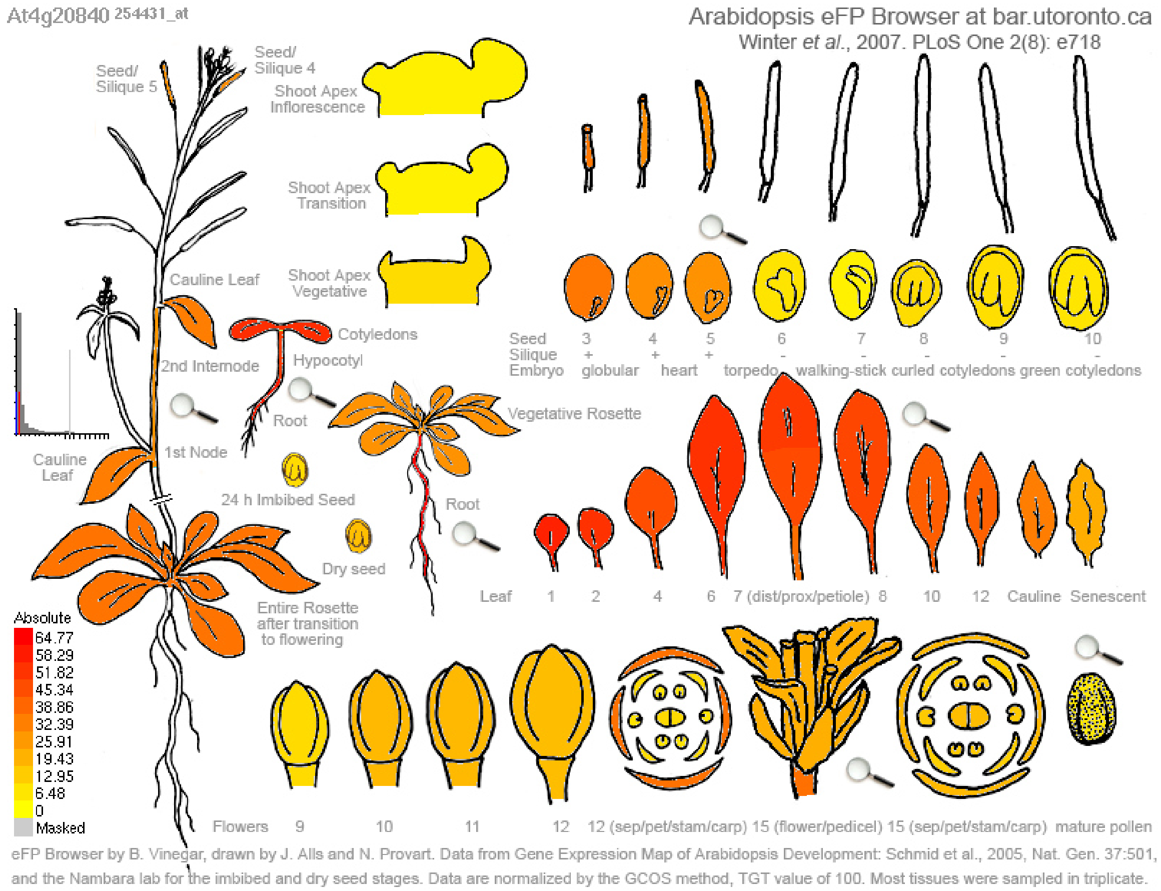

Figure S2. ***OGOX2*** **(At4g20840) is expressed in leaves.**
Data are from eFP Browser.

A

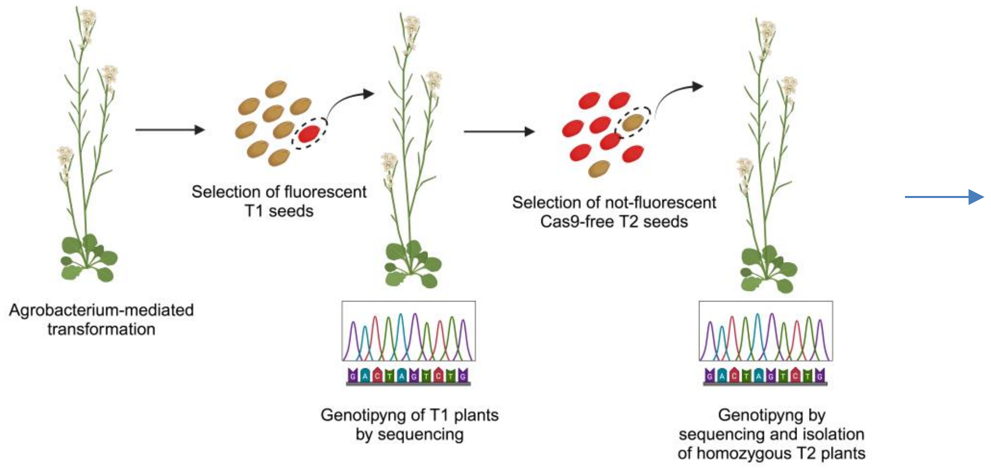

T1 line	*OGOX1 mutation*	*OGOX2 mutation*
**#1**	**X**	**X**
#2	WT	WT
#3	WT	WT
**#4**	**X**	**X**
**#5**	**X**	**WT**
**#7**	**WT**	**X**
**#8**	**X**	**WT**
**#9**	**WT**	**X**
**#10**	**X**	**WT**
#11	WT	WT
**#12**	**X**	**WT**
#13	WT	WT

B

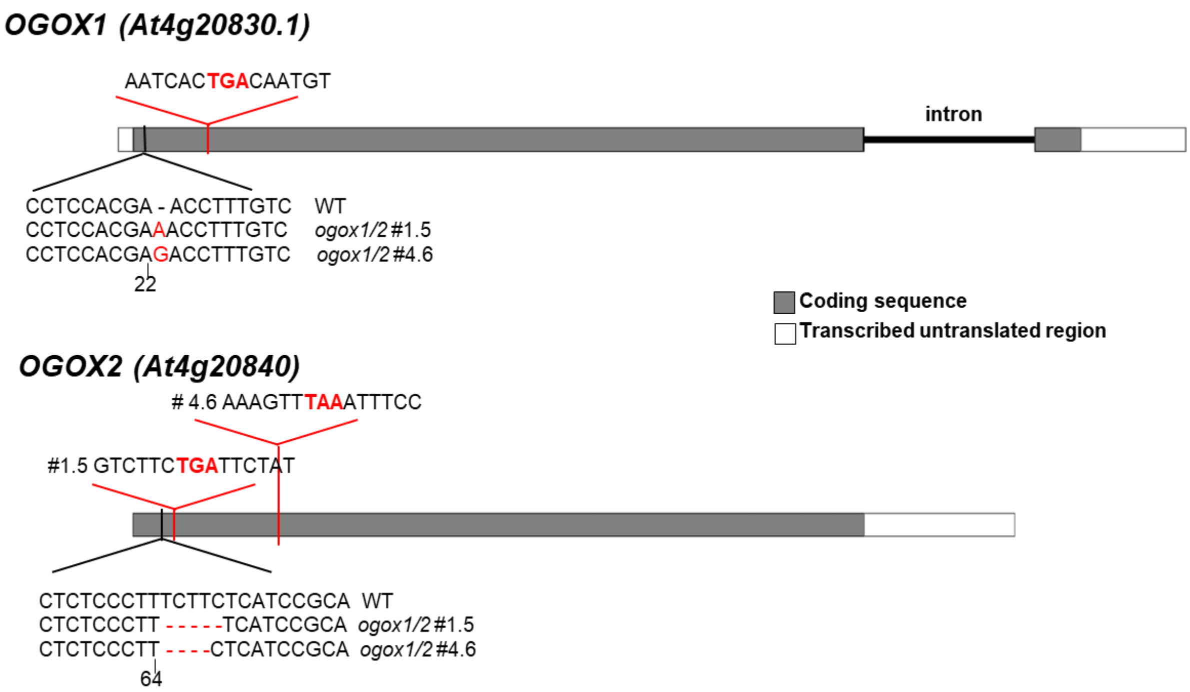

Figure S3. Construction strategy for *OGOX1* and *OGOX2* genes editing by CRISPR/Cas9 via *Agrobacterium*-mediated transformation of Arabidopsis and mutant screening approaches.
A. Sequential steps followed for the selection of CRISPR-Cas9 edited ogox1 and ogox2 mutants. Twelve T1 transformed seeds (screened based on the presence of the Fast-Red selection marker) were selected and grown. Mutations were found by sequencing in seven plants. Two plants, #1 and #4, showed a mutation in both *OGOX1* and *OGOX2*.
B. Schematic representation of the position of the frameshift mutations introduced by gene editing in both *OGOX1* and *OGOX2* in the double mutants ogox1/2 lines #1.5 and #4.6.
The single-nucleotide insertions after nucleotide 22 in *OGOX1* and the four- or five-nucleotide deletions after nucleotide 64 are all frameshift mutations that introduce stop codons (shown above in bold red).

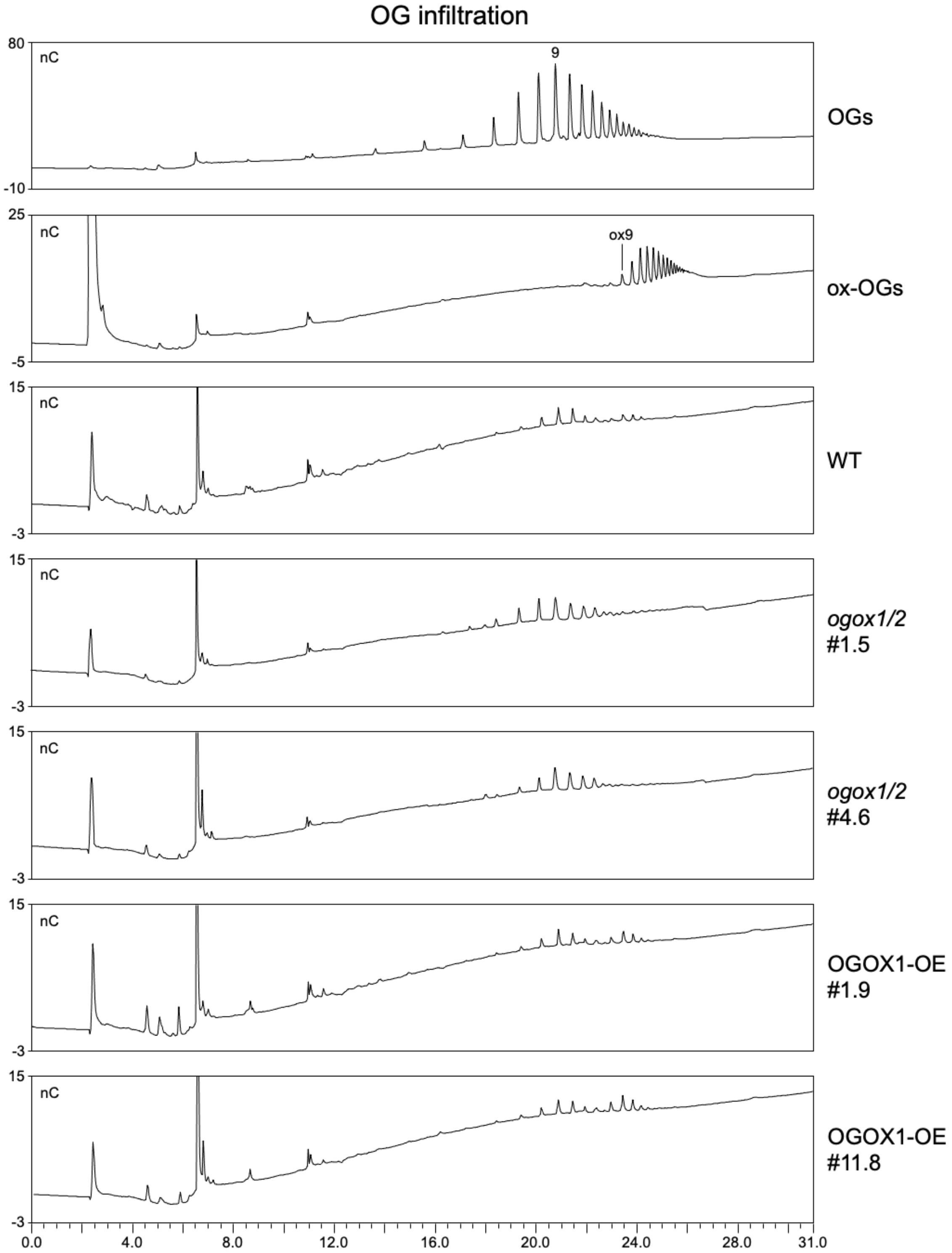

Figure S4. Complete HPAEC-PAD chromatographic profiles of chelating agent-extracted oligosaccharides (ChASS) from total cell wall preparations (AIS) of WT, *ogox1/2* and OGOX1-OE leaves infiltrated with OGs. OGs and oxidized OGs indicated by numbers corresponding to the degree of polymerization (DP). OG and ox-OG preparations were used as standards. Profiles of OGs and ox-OGs (DP 9-11) from retention times 21 to 26 min are shown in Fig. 3A.

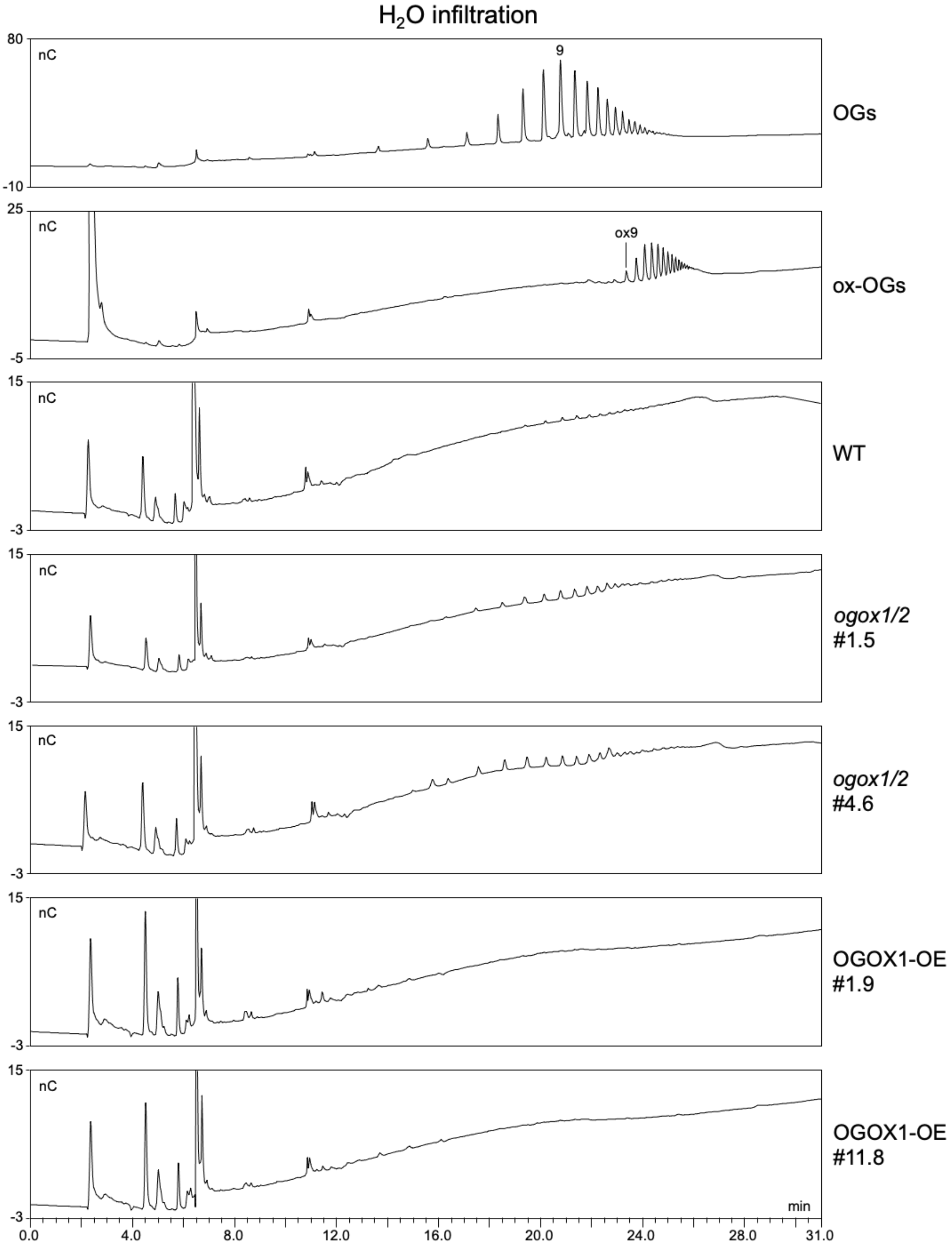

Figure S5. Complete HPAEC-PAD chromatographic profiles of chelating agent-extracted oligosaccharides (ChASS) from total cell wall preparations (AIS) of WT, *ogox1/2* and OGOX1-OE leaves infiltrated with H_2O.OGs and oxidized OGs indicated by numbers corresponding to the degree of polymerization (DP). OG and ox-OG preparations were used as standards. Profiles of OGs and ox-OGs (DP 9-11) from retention times 21 to 26 min are shown in Fig. 3B

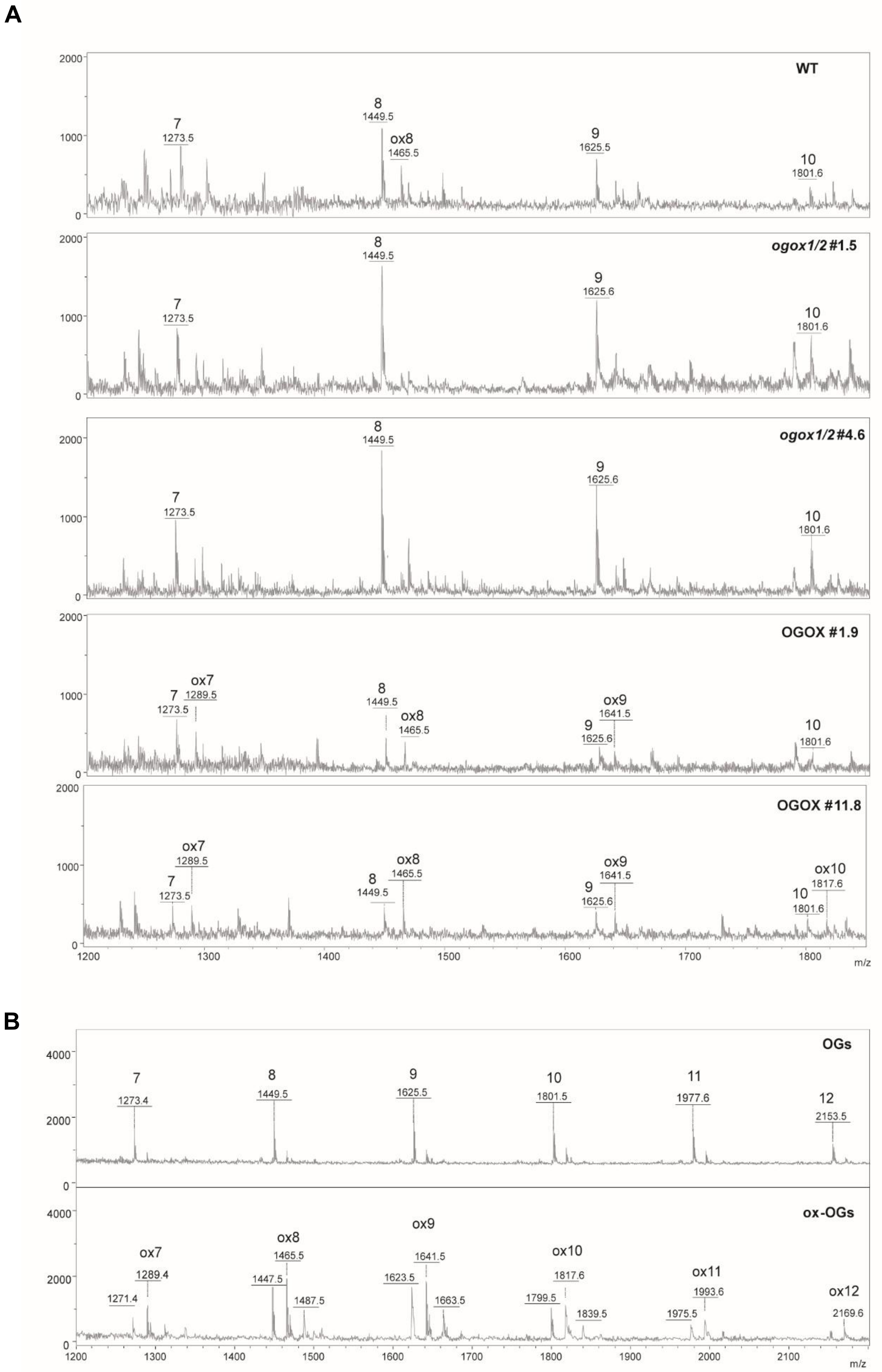

Figure S6 MALDI-TOF full-scan mass spectrum (MS) of chelating agent-extracted oligosaccharides (ChASS) from total cell wall preparations (AIS) of WT, *ogox1/2* and OGOX1-OE adult leaves infiltrated with OGs. A) Full-scan MS of OG-containing fractions (ChASS) from OG-infiltrated leaves, analyzed by HPAEC-PAD as illustrated in Figure 3**. B.** Full-scan MS of OG and ox-OG standards (0.5 µg each). In both figures, red numbers above peaks represent the degree of polymerization (DP) of the individual OG oligomers; the ox"number" indicates the DP of the oxidized oligomers, characterized by an increased mass (+16 m/z) compared to their unmodified counterparts. The main ions detected in the analysis of OGs are identified as Na^+ adducts, based on their m/z values (22 m/z). Additionally, secondary peaks correspond to $2Na^+$ adducts and m/z values resulting from water loss (-18 m/z).

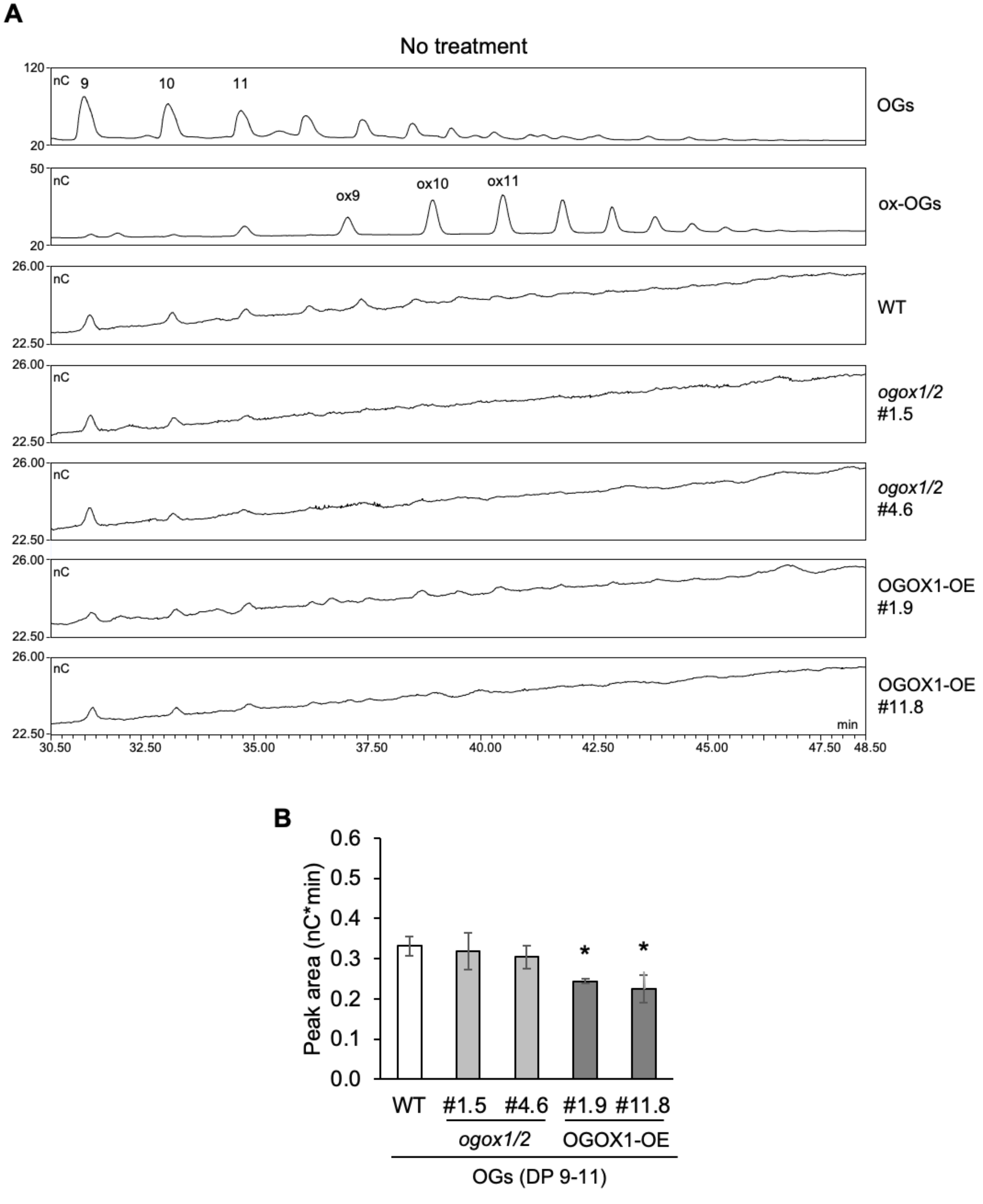

Figure S7. HPAEC-PAD analyses of chelating agent-extracted oligosaccharides (ChASS) from total cell wall preparations (AIS) of WT, *ogox1/2* and OGOX1-OE leaves. In the chromatographic profiles (A), OGs and oxidized OGs (ox-OGs) are indicated by numbers corresponding to the degree of polymerization (DP). Graph in B shows the sum of peak areas of OGs (DP 9-11) as seen in the chromatographic profiles. OG and ox-OG preparations of DP 9-16 were used as standards.

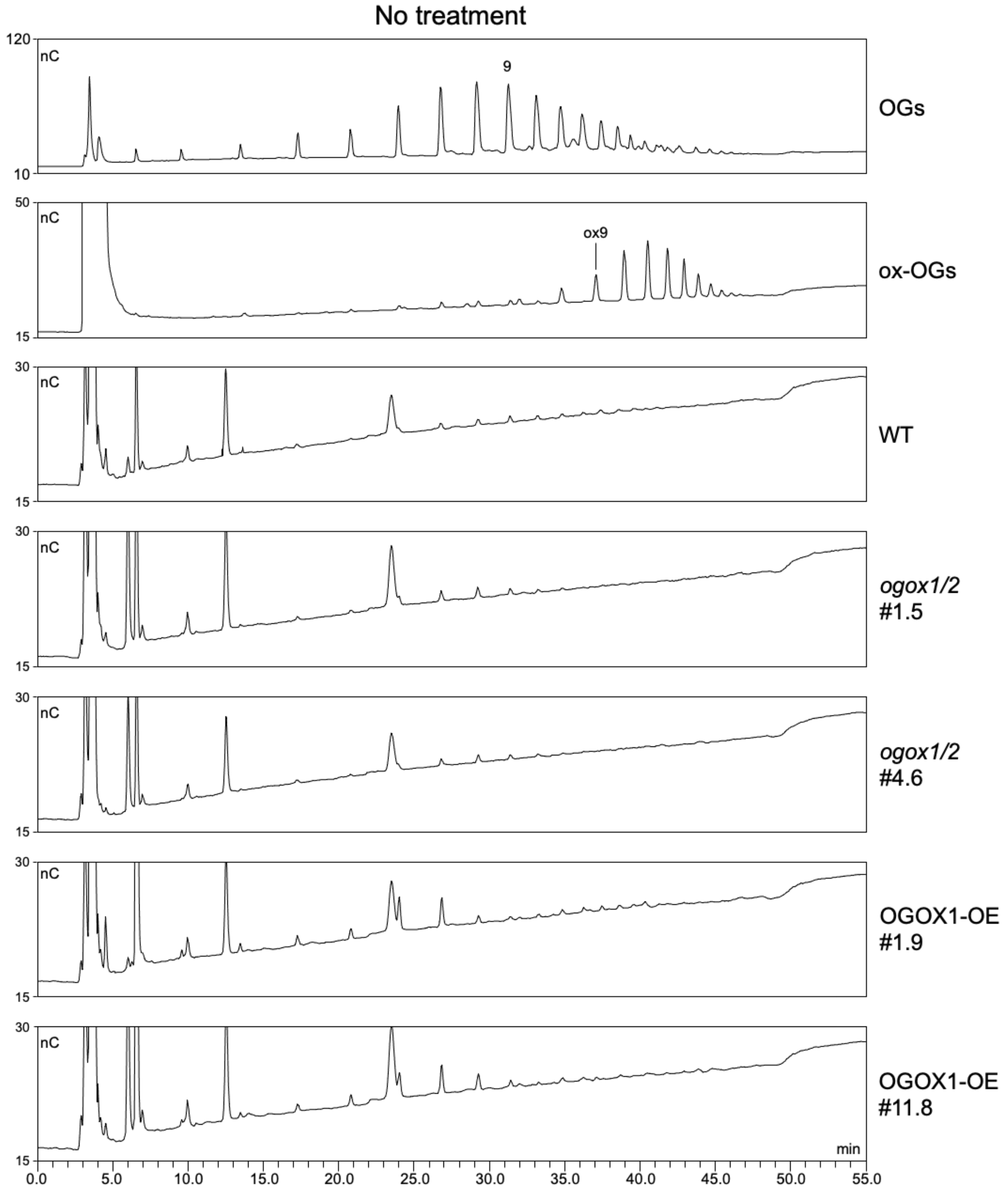

Figure S8. Complete HPAEC-PAD chromatographic profiles of chelating agent-extracted oligosaccharides (ChASS) from total cell wall preparations (AIS) of WT, *ogox1/2* and OGOX1-OE leaves. OGs and oxidized OGs indicated by numbers corresponding to the degree of polymerization (DP). OG and ox-OG preparations were used as standards. Profiles of OGs and ox-OGs (DP 9-11) from retention times 21 to 26 min are shown in Figure S7.

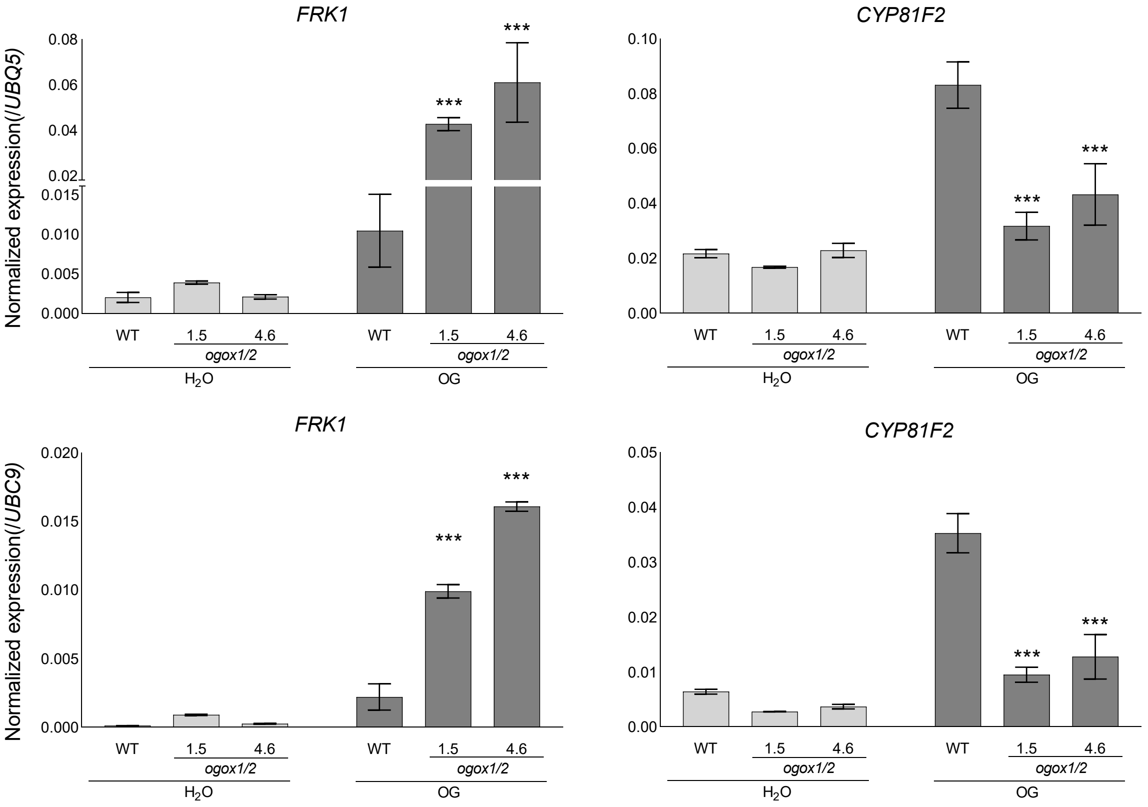

Figure S9. Quantitative RT-PCR analysis of *FRK1* and *CYP81F2* expression induced by OGs using two different housekeeping genes as reference (*UBQ5* and *UBC9*). Expression was analyzed in rosette leaves from four-week-old plants of *Arabidopsis* Col-0 WT and CRISPR Cas-deleted *ogox1/ogox2* (lines #1.5 and #4.6) plants at 1 h post-infiltration with OGs (60 µg/ml) or water as control. *UBQ5* (top panels) and *UBC9* (bottom panels) transcript levels were used for normalization. The mean of two biological replicates (± SD) is shown. Samples are the same as in main Figure 4. Asterisks indicate statistically significant differences of mutants compared to WT plants subjected to the same treatment according to Student's t-test. (* $p < 0.05$; ** $p < 0.01$; *** $p < 0.001$).

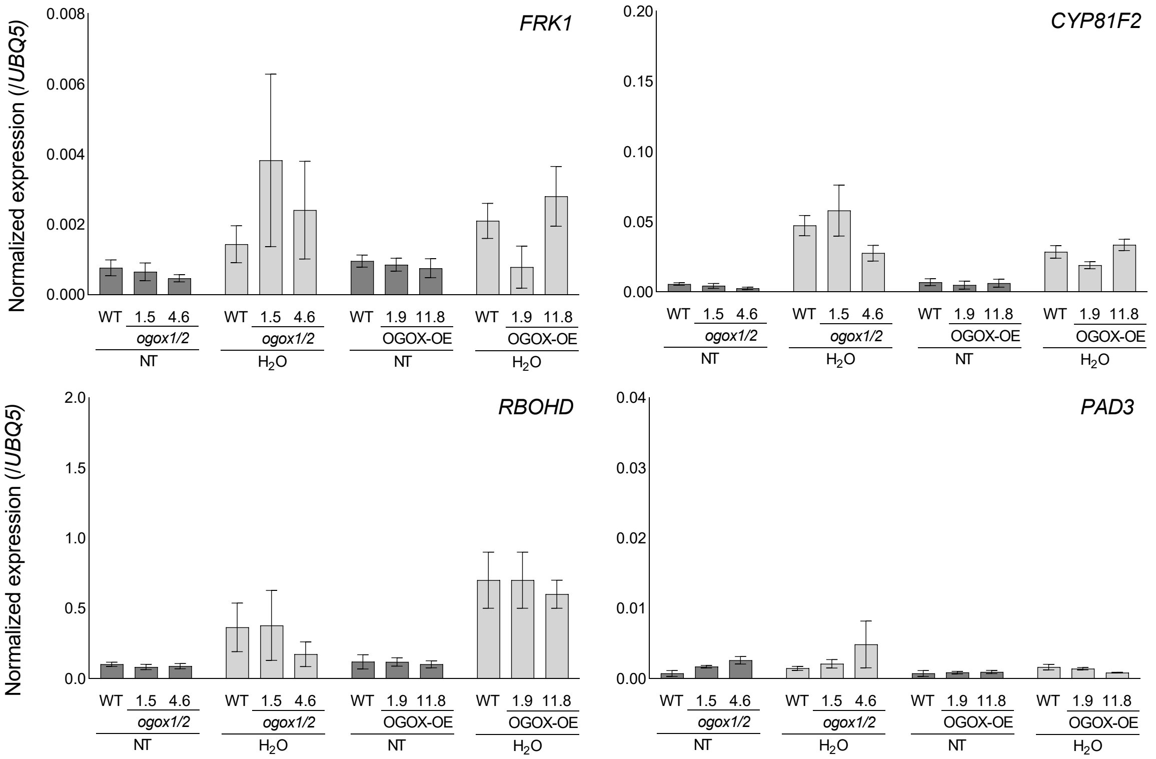

Figure S10. Analysis of defense-related gene expression in untreated non-infiltrated plants and plants infiltrated with water. Expression was analyzed by quantitative RT-PCR in rosette leaves of untreated plants (NT) and plants at 1 h post-infiltration with water (H_2O). Four-week-old plants of Col-0 (WT), CRISPR Cas-deleted *ogox1/2* plants (lines #1.5 and #4.6) and OGOX1-overexpressing plants (OGOX1-OE, lines #1.9 and #11.8) were used. *UBQ5* transcript levels were used for normalization. The mean of three biological replicates (± SD) is shown.

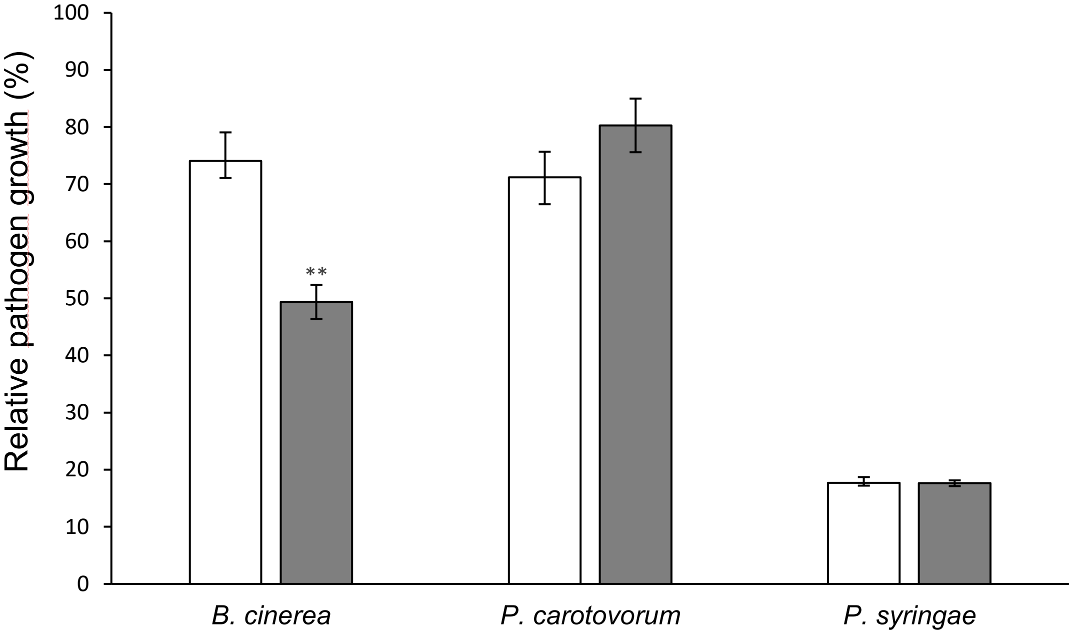

Figure S11. OG-to-growth conversion by *B. cinerea*, *P. carotovorum* and *P. syringae*. Relative growth of *B. cinerea*, *P. carotovorum* and *P. syringae* DC3000 in minimal medium supplemented with 0.15% (w/v) OGs (white bars) and 0.15% (w/v) oxOGs (grey bars) after 24 hour of incubation. Values are the ratio to the maximal growth observed in the same minimal medium supplemented with 0.15% (w/v) D-glucose. Values are mean ± SD (N=3). Asterisks indicate statistically significant differences according to Student's t test (**, $p < 0.005$).

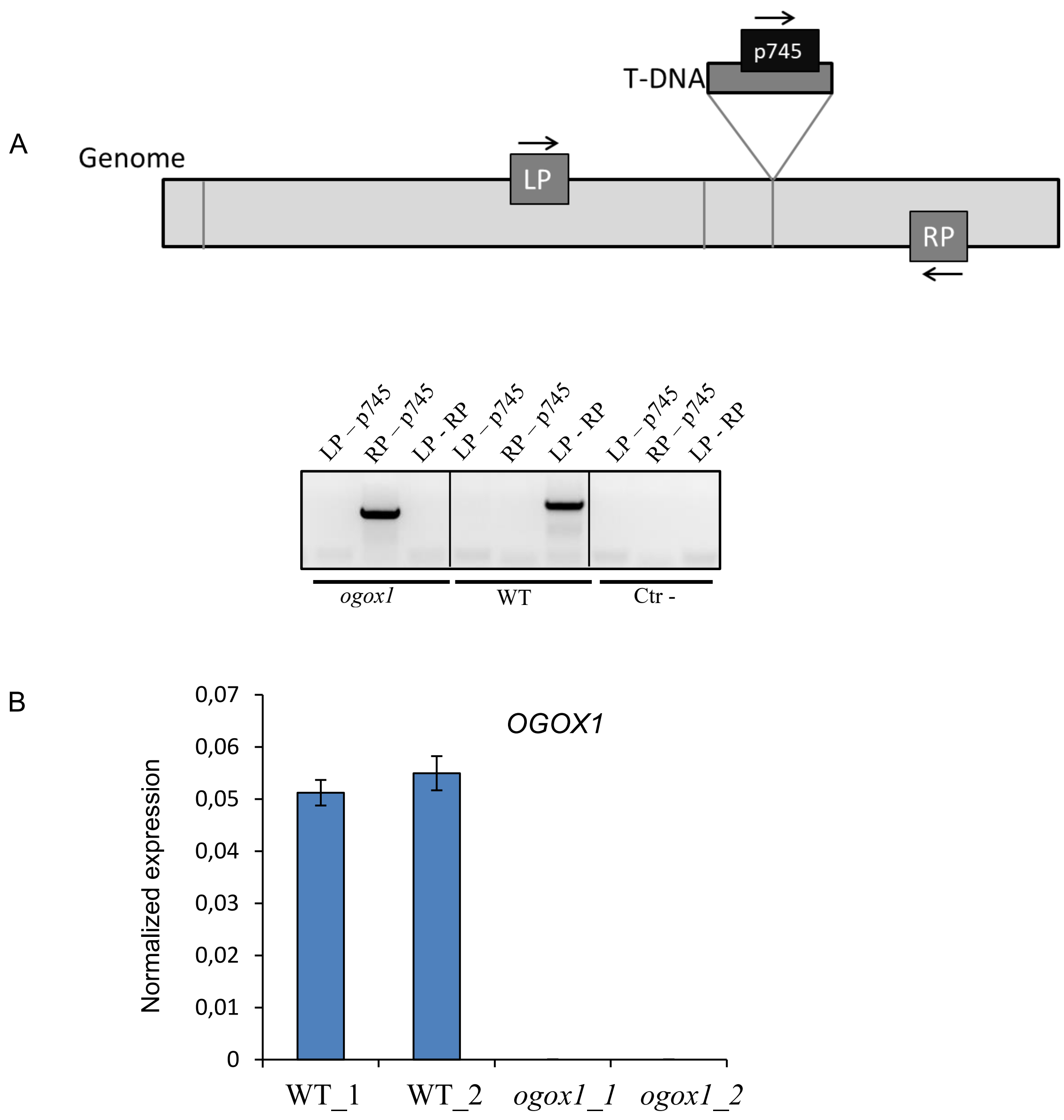

Figure S12. Characterization of the T-DNA insertional mutants *ogox1.*
A. Schematic representation (top) of T-DNA insertion in *ogox1* mutant (WISCDSLOX432E05) and PCR on genomic DNA (bottom) from WT and *ogox1* plants using the primer pairs specific for the WT gene (LP+RP) or for the T-DNA insertion (LP/RP+ p745). **B.** Expression of *OGOX1* transcripts analysed by quantitative qRT-PCR in adult leaves of Arabidopsis Col-0 WT and *ogox1* mutant. UBQ5 transcript levels were used for normalization. Values of two biological replicates are shown (± SD).

A

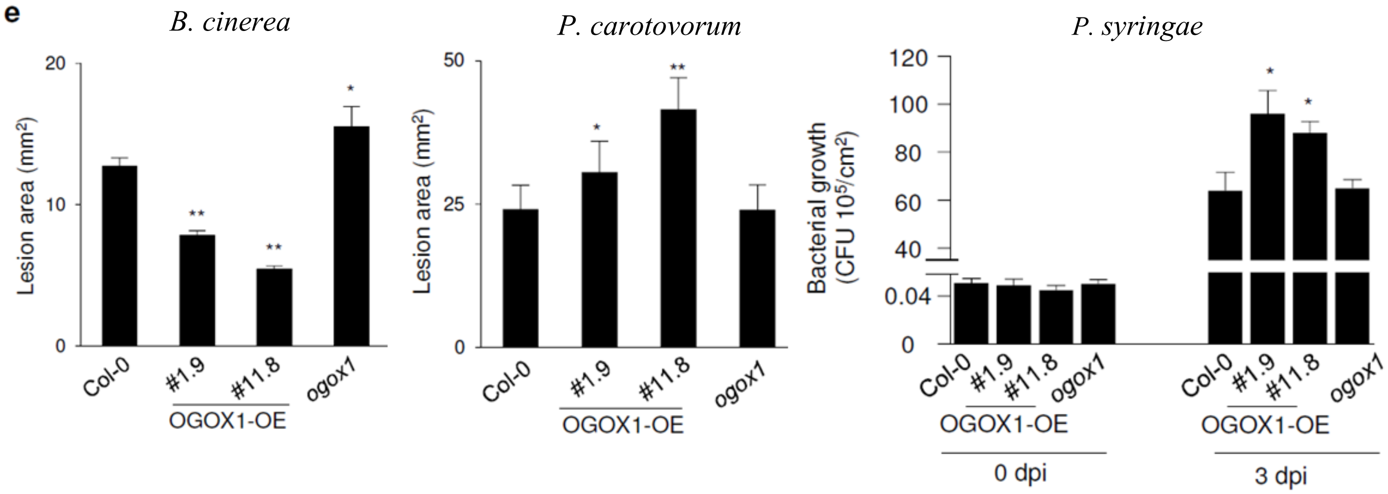

B

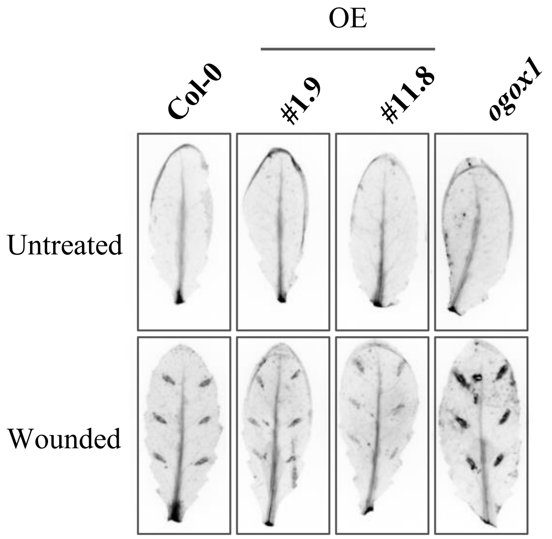

Figure S13. Analysis of pathogens resistance and hydrogen peroxide accumulation inded by mechanical damage in the *ogox1* null mutant and in the OGOX1-overexpressing lines.
A. Lesion areas produced by *B. cinerea and P. carotovorum* were quantified at 48 and 16 hpi, respectively, using the ImageJ software. *Pst* DC3000 spread was quantified 72 hpi. Asterisks indicate statistically significant differences of mutants compared to WT according to Student's t-test (*$p < 0.05$; **$p < 0.01$). **B.** Leaves of four-week-old plants were wounded with knurled-tip tweezers, excised after 1 h and subjected to DAB staining for 12 h. At least six wounded leaves from three independent plants were used for each genotype. Untreated leaves were used as controls. The experiment was repeated three times with similar results; a representative experiment is shown. In both A and B, OGOX1-overexpressing (OGOX1-OE; two independent lines) plants were used in parallel.

Table S1. Primers used in this work

Gene expression analysis	
CYP81F2-Fw	5’-ATCGCCCATTCCAATGTTA-3’
CYP81F2-Rv	5’-AAATGGAGAGAGAGCAACACAATG-3’
PAD3-Fw	5’-CCGGTGAATCTTGAGAGAGCC-3’
PAD3-Rv	5’-GATCAGCTCGGTCATTCCCC-3’
FRK1-Fw	5’-TGCACTTACCCTCCTTCG-3’
FRK1-Rv	5’-GACAGTAGAAGCCGGTTGGT-3’
RBOHD-Fw	5’-CTAAAGACTTCGCTGACC-3’
RBOHD-Rv	5’-CTTCTTCTGTTACTCGCC-3’
UBQ5-Fw	5’-GTTAAGCTCGCTGTTCTTCAGT-3’
UBQ5-Rv	5’-TCAAGCTTCAACTCCTTCTTTC-3’
UBC9- Fw	5’-TGCCTCGACATCTTGAAGGA-3’
UBC9-Rv	5’-TACTTTTGGGTCCAGGTCCG-3’
Genotyping WISCDSLOX432E05	
P745	5’-AACGTCCGCAATGTGTTATTAAGTTGTC-3’
OGOX1 LP	5’-CGAATTCGAAACCACTGATTG-3’
OGOX1 RP	5’-ATCACGGAGATTCGACATGTC-3’
Genotyping CRISPR OGOX1/2	
830 CRISPR fw	5’-ACACGTCTTCTTCTCCCTGC-3’
830 CRISPR rev	5’-GAGACCATCGTAGTCATGGC-3’
840 CRISPR fw	5’-TCTTCTCTGTTTAGTGGTGG-3’
840 CRISPR rev	5’-GGAGATTCGACATGTCGAGT-3’
Golden Gate Cloning	
sgRNA OGOX1/OGOX2	5’-TGTGGTCTCAATTGAAGTGACGGCGGCGCTGACGGTTTAAG AGCTATGCTGGAA-3’
sgRNA OGOX1a	5’-TGTGGTCTCAATTGTGCTTACATCCGCAATGCGGTTTAAGAG CTATGCTGGAA-3’
sgRNA OGOX1b	5’-TGTGGTCTCAATTGGGACAGAGACAAAGGTTCGGTTTAAGAG CTATGCTGGAA-3’
sgRNA OGOX2a	5’- TGTGGTCTCAATTGTGCTTACATTCGAAATGGCGTTTAAGA GCTATGCTGGAA-3’
sgRNA OGOX2b	5’-TGTGGTCTCAATTGTGCTGCGGATGAGAAGAAAGTTTAAGAG CTATGCTGGAA-3’
sgRNA Reverse	5’-TGTGGTCTCAAGCGAAAAAAAGCACCGACTC-3’
USER Cloning	
OGOX1gDNA Fw	5’-TGGGATTTACGATTACTCTTGC-3’
OGOX1 gDNA Rv	5’-TCGGAGAAACACTTGAGGAAA-3’
OGOX1 gDNA ter Fw	5’- TCTTCTTCTCCCTGCGGTTA-3’
OGOX1 gDNA ter Rv	5’- AGCTGGTTTTGGCGTAGATG-3’
OGOX2 gDNA Fw	5’- CATCGGTCGTTACGGTGTTT-3’
OGOX2 gDNA Rv	5’- GGTGGATGTGTGATTCGGTA-3’

POGOX1-GFP Fw	5’- GGCTTAAUGTGTACTGTGTACATCCCTA-3’
POGOX1-GFP Rv	5’-ACCATTUGAGAGAAAGATGAGAGAGGT-3’
OGOX1 CDS Fw	5’- ATCGAACUCTGTATACAACTCTTTCCTC-3’
OGOX1 CDS Rv	5’- GGTTTAAUCGTTGTAATGAGATTCGTTG-3’
N-ter GFP Fw	5’-AAATGGUGAGCAAGGGCGAGGAGCTGT-3’
N-ter GFP Rv	5’- AGTTCGAUGATCTAATAGCCGCGTTTTT-3’
POGOX2-OGOX2 Fw	5’-GGCTTAAUGTTTCTTGATCGGAATCTCGA-3’
POGOX2-OGOX2 Rv	5’- ACCGATGCCUTGTAAGCACGGTAGGTATA-3’
OGOX2 ter Fw	5’-AGTAAGCAUGGAGTTTGGACTAGACCAA-3’
OGOX2 ter Rv	5’- GGTTTAAUCTTTCTTCGGAAGTAGTGCT-3’
C-ter GFP Fw	5’-AGGCATCGGUGAGCAAGGGCGAGGAGCTGT-3’
C-ter GFP Rv	5’-ATGCTTACUGTACAGCTCGTCCATGCCG-3’
POGOX1::GUS cassette	
BamHI-POGOX1 Fwd	5’-CGCGGATCCGTTTGATTTTTTTTTTTTTTTTTGC-3’
BamHI-POGOX1 Rev	5’-CGCGGATCCTGTTCTTTGTACTTATCCGTGT-3’

Table S2. sgRNAs targeting sequences and Level 1 plasmids

sgRNA	Sequence	Plasmid (Position)
sgRNA OGOX1/OGOX 2	GAAGTGACGGCGGCGCTGACG	pICH4775 1 (Position 3)
sgRNA OGOX1a	GTGCTTACATCCGCAATGCG	pICH4776 1 (Position 4)
sgRNA OGOX1b	GGGACAGAGACAAAGGTTCG	pICH4777 2 (Position 5)
sgRNA OGOX2a	GTGCTTACATTCGAAATGGC	pICH4778 1 (Position 6)
sgRNA OGOX2b	GTGCTGCGGATGAGAAGAAA	pICH4779 1 (Position 7)
